# Supplementary material for: Knowledge of postpartum complications and associated factors among women who gave birth in the last 12 months in Arba Minch Town, Southern Ethiopia, 2019: A community-based cross-sectional study
Source: PLoS One. 2023 Feb 6;18(2):e0281242. doi: 10.1371/journal.pone.0281242 (PMC9901784; doi:10.1371/journal.pone.0281242)
Supplement: S1 File — (DOCX) [file pone.0281242.s001.docx]

**English version questionnaire**

Good Morning/Afternoon, My name is _______________________________. I am a trained data collector for the research; **knowledge on postpartum complications and factors among mothers who gave birth in the last 12 months in Arba Minch town** which is being conducted by **Godana Yaya** who is a Master of Clinical midwifery student in **Arba Minch University**. Your kebele and your house are selected as a **matter of chance**; and nothing special is attached to it. If you participate in the study, there will **not be any harm** to you or your family. Your name and other personal identifiers will not be recorded on data collection form and the **information that you give us will be kept confidential**. The information you provide will not be used for another purpose than this study. For this interview, there will **not be an immediate benefit in terms of money**; rather the information you provide will help the government and concerned bodies **to improve mothers’ knowledge on postpartum complications** which can also benefit you and your community. Your participation is **voluntary** and you have the right to stop the interview at any time. Your decision about not to participate is respected.

However, your real honest participation will contribute to generate information that could be used for better reproductive health program design and improvement of the reproductive health service. I would greatly appreciate your participation in this interview. It will take about **10 – 15 minutes.**

Would you be willing to participate in this interview?

1. Yes (Continue)

2. No (Thank you)

Date & signature .

Name of Supervisor .

|  | **Questions** | **Responses** | | **Skip to** | |
| --- | --- | --- | --- | --- | --- |
|  | **Socio-demographic characteristics** | |  | |  |
|  | How old are you? | __________age in years | |  | |
|  | What is your religion? | 1. Orthodox 2. Protestant 3. Muslims 4. Catholic 5. Other (specify)________ | |  | |
|  | What is your marital Status? | 1. married 2. widowed 3. divorce 4. Not married 5. Other (specify)------------------ | |  | |
|  | To which ethnic group do you belong to? | 1. Gamo 2. Zeyissie 3. Gofa 4. Derashe 5. Konso 6. Gurage 7. Amhara 8. Other, specify_________ | |  | |
|  | What is your occupation? | 1. Government employee 2. Merchant 3. House wife 4. Other, specify-____ | |  | |
|  | What is your monthly income? | ________(in birr) | |  | |
|  | What is your educational  Status? | 1. Didn’t attend school 2. Primary school 3. Secondary school and above | |  | |
|  | Do you have access for mass media? | 1. Yes 2. No | |  | |
|  | If yes, what do you use? | 1. Radio 2. TV 3. Newspaper 4. Other (specify)s______ | |  | |
|  | **History of obstetric characteristics and health service utilization** | |  | |  |
|  | How many times in total you gave birth? | _________(in Number) | |  | |
|  | Did you have ANC follow up for your last pregnancy? | 1. Yes 2. No | | If no, skip to Q.15 | |
|  | If yes, how many times you attended? | - 1. Once   2. Two times   3. Three times   4. Four and above | |  | |
|  | Where did you attend your ANC visit? | - 1. Health center   2. Hospital   3. Private clinic   4. Other……. | |  | |
|  | Did you informed on pregnancy related problems during your follow up? | - 1. Yes   2. No | |  | |
|  | Where did you give your last birth? | - 1. At health institution   2. Home | |  | |
|  | Who is final decision maker on health service utilization? | - 1. Self   2. Husband or other family member | |  | |
|  | Do you assume that; there is enough access of health facility in your town? | - 1. Yes   2. No | |  | |
|  | **Knowledge about postpartum complication** | |  | |  |
|  | Have you information about postpartum complications that can happen after child birth? | 1. Yes 2. No | If no, skip to Q. 22 | |  |
|  | If yes, where did you get the information? | 1. During ANC follow up 2. Meeting on maternal health care 3. Mass media 4. School 5. Others…….. |  | |  |

|  | Which complication do you know? Spontaneously mothers should mention & you circle it. | |  |
| --- | --- | --- | --- |
| 1. Sever vaginal bleeding after child birth? 2. Foul-smelling vaginal discharge after child birth? 3. Hypertension after child birth that related to pregnancy? 4. Breast complication after child birth? 5. Convulsions after child birth? 6. Blurred vision and weakness? 7. Psychoses and blues? 8. Others | |  |  |
|  |  |  |  |
|  |  |  |  |
|  |  |  |  |
|  |  |  |  |
|  |  |  |  |
|  |  |  |  |
|  |  | ………….. |  |
|  | Where do you go if these problems happen? | 1. Health institutions 2. Traditional healers 3. Other(specify)_____________ |  |
|  | If ‘No’ on question Number ‘18’ what was the reason? | - 1. I didn’t expect anything to occur   2. I never heard   3. Other……………. |  |

Thank you!!!

**Amharic version questionnaire**

ደህና አደሩ / ዋሉ፣ስሜ _______________________________ ነው፡፡ እኔ በአርባምንጭ ከተማ ውስጥ ባለፉት 12 ወራት ከወለዱ እናቶች መካከል በድህረ ወሊድ ችግሮች ላይ የሴቶች ዕውቀት እና ተዛማች ምክንያቶች ላይ **በአርባምንጭ ዩኒቨርሲቲ ውስጥ የክሊኒካል ሚዲዋይፌሪ የጤና ሙያ ዘሪፍ የ3ኛ ዓመት የማስተርስ ተማሪ በሆኑት መ/ር ጎዳና ያያ** በሚጠና ምርምር የሰለጠንኩ የመረጃ ሰብሳቢ ነኝ። ቀበሌዎ እና ቤትዎ እንደ ዕድል ጉዳይ ተመርጠዋል ፡፡ እና ምንም ልዩ ነገር አልተያያዘለትም። በጥናቱ ውስጥ የሚሳተፉ ከሆነ በእርስዎም ሆነ በቤተሰብዎ ላይ ምንም ዓይነት ጉዳት አይኖርም ፡፡ ስምዎ እና ሌሎች የግል መለያዎች በመረጃ አሰባሰብ ቅፅ ላይ አይመዘገቡም እናም የሰጡን መረጃ በምስጢር ይጠበቃል ፡፡ የሚሰጡት መረጃ ከዚህ ጥናት ውጭ ለሌላ አገልግሎት አይውልም ፡፡ ለዚህ ቃለ መጠይቅ ፣ በገንዘብ ረገድ አፋጣኝ ጥቅም አይኖርም ፣ ይልቁንም የሚሰጡት መረጃ መንግስት እና ጉዳዩ የሚመለከታቸው አካላት እርስዎ እና ማህበረሰብዎም ሊጠቅሙ የሚችሉ የድህረ ወሊድች ግሮች ላይ የእናቶችን እውቀት ለማሻሻል ነዉ፡፡ተሳትፎዎ በፍቃደኝነት ነው እናም በቃለ መጠይቁ በማንኛውም ጊዜ ለማቆም መብት አልዎት ፡፡ ላለመሳተፍ ያደረጉት ውሳኔ ይከበራል ፡፡ ሆኖም እውነተኛ ቅንነትዎ ለተሻለ የስነ-ተዋልዶ ጤና መርሃ ግብር ዲዛይን እና ለመራቢያ ጤና አገልግሎት መሻሻል ሊያገለግል የሚችል መረጃን ለማመንጨት አስተዋፅኦ ያደርጋል ፡፡ በዚህ ቃለ-መጠይቅ ላይ ተሳትፎዎን በእጅጉ አደንቃለሁ ፡፡ ከ 10 - 15 ደቂቃዎች ይወስዳል ፡፡

በዚህ ቃለ መጠይቅ ውስጥ ለመሳተፍ ፈቃደኛነዎት?

1. አዎ (ይቀጥሉ)

2. አይ (አመሰግናለሁ)

ቀን እና ፊርማ .

የተቆጣጣሪ ስም .

|  | **ጥያቄዎች** | | | **ምላሾች** | **እለፍ** | | | |  |
| --- | --- | --- | --- | --- | --- | --- | --- | --- | --- |
| ተ/ቁ | **ክፍል አንድ፡ ማህበራዊና የስነ ሕዝብ መረጃ መጤይቆች** | | |  |  | | | |  |
|  | እድሜዎ ስንት ነው? | | | ………………….(በዓመት) |  | | | |  |
|  | ሃይማኖትዎ ምንድን ነው? | | | - 1. ኦርቶዶክስ   2. ፕሮቴስታንት   3. ሙስሊም   4. ካቶሊክ   5. ሌላ(ይግለጹ) ___ |  | | | |  |
|  | የጋብቻ ሁኔታዎ ምን ይመስላል ? | | | 1. ባለትዳር 2. ባለቤቷ የሞተባት 3. የተፋታች 4. ያላገባች 5. ሌላ (ይግለጹ) -------- |  | | | |  |
|  | ብሔርዎ ምንድን ነዉ ? | | | 1. ጋሞ 2. ዘይሴ 3. ጎፋ 4. ደራሼ 5. ኮንሶ 6. ጉራጌ 7. አማራ 8. ሌላ(ይግለጹ ,………… |  | | | |  |
|  | | ሥራዎ ምንድን ነው? | 1. ነጋዴ 2. የመንግስት ሰራተኛ 3. የቤት እመቤት 4. ሌላ, ይግለጹ -____ | | | |  | | |
|  | | የወር ገቢዎ በብር ምንያህል ነው? | ______ (በኢትዮጵያብር) | | | |  | | |
|  | | የትምህርት ደረጃ | 1. ያልተማረች 2. የመጀመሪያ ደረጃ ት/ቤት የተማረች 3. ሁለተኛ ደረጃ እና ከዝያ በላይ የተማረች | | | |  | | |
|  | | የመገናኛ ብዙሃን መዳረሻ አለዎት? | 1. አዎ  2. አይ | | | |  | | |
|  | | አዎ ከሆነ, ምንይ ጠቀማሉ? | 1. ሬዲዮ  2. ቴሌቪዥን  3. ጋዜጣ  4. ሌላ (ይግለጹ)…………. | | | |  | | |
| **ክፍል ሁለት፡- የወሊድ ባህሪያት ታሪክ እና የጤና አጠቃቀም** | | | | | | | | | |
|  | | በአጠቃላይ ስንት ጊዜ ወልደዋል? | .(በቁጥር) | | |  | | | |
|  | | ባለፈው እርግዝና ወቅት የቅድመ ወሊድ ምርመራ ክትትል አድርገዉ ያዉቃሉ ? | 1. አዎ 2. አይ | | | አይ ከሆነ, ወደ ጥያቄ 15. ይዝለሉ | | | |
|  | | አዎ ከሆነ, የቅድመ ወሊድ ምርመራዉን ስንት ጊዜ ተከታትለዋል ? | 1. አንድ ጊዜ  2. ሁለት ጊዜ  3. ሦስት ጊዜ  4. አራት እና ከዚያ በላይ | | |  | | | |
|  | | ክትትል ያደረጉት የት ነበር? | 1. ጤናጣቢያ  2. ሆስፒታል  3. የግልክሊኒክ  4. ሌላ…… | | |  | | | |
|  | | በሚከታተሉበት ወቅት ከእርግዝና ጋር ተያያዥነት ያላቸውን ችግሮች በተመለከተ መረጃ አግኝተዋል? | 1. አዎ  2. አይ | | |  | | | |
|  | | የመጨረሻ ልጅሽን የት ወለድሽ ? | 1. በጤና ተቋም  2. ቤት | | |  | | | |
|  | | በጤና አገልግሎት አጠቃቀም የመጨረሻ ውሳኔ ሰጭ ማን ነው? | - 1. እራሰ   2. ባል / ቤተሰብ | | |  | | | |
|  | | በከተማዎ ውስጥ በቂ የጤና ተቋም አለ፡ ብለዉ ያስባሉ? | 1. አዎ  2. አይ | | |  | | | |
| **ክፍል ሶስት፡- በድህረ ወሊድ ወቅት የሚፈጠሩ የጤና ችግሮች እውቀት** | | | | | | | | | |
|  | ከወለድ በኋላ ሊከሰቱ የሚችሉ ችግሮች መረጃ አለዎት? | | 1. አዎ  2. አይ | | | | | አይከሆነወደጥያቄ 22 ይዝለሉ | |
|  | አዎ ከሆነ መረጃውን ከየት አገኙት? | | 1. በቅድመ ወልድ ክትትል ወቅት 2. በእናቶች ጤና ዙርያ የሚደረግ ዉይይት ወቅት 3. ከመገናኛ ብዙኃን 4. ትምህርት ቤት 5. ሌሎች …… .. | | | | |  | |
|  | ከወለድ በኋላ ሊከሰቱ የሚችሉ ችግሮችን ይጥቀሱ………..  (እያከበቡ መጤየቆን ይቀጥሉ) | | 1. ከፍተኛ የደም መፍሰስ 2. ሽታ ያለዉ ፈሳሽ መፍሰስ 3. የአእምሮ ሕመም/ መታወክ 4. ከእርግዝና ጋር በተዛመደ ከወሊድ በኋላ የደም ግፊት መጨመር 5. የደበዘዘ ዕይታ እና ድካም 6. እንደ ሚጥል በሽታ መንፈራፈር 7. ልጅ ከወለዱ በኋላ የጡት ህመም ችግር 8. ሌላ ......... | | | | |  | |
|  | እነዚህ ችግሮች ከተከሰቱ የት ይሄዳሉ? | | 1. ወደ ጤና ተቋም  2. ወደ ባህላዊ ፈዋሾች  3. ሌላ (ይግለጹ) _____________ | | | | |  | |
|  | በጥያቄ ቁጥር 18 'አይ' ከሆነ 'ምክንያቱ ምንድን ነበር? (የመለሱትን ብቻ ይጻፉ ወይም ይክበቡ) | | 1. የሆነ ነገር ይከሰታል ብዬ አልጠብቅም  2. ሰምቼ አላዉቅም  3. ሌላ ………. | | | | |  | |

አመሰግናለሁ !!!
