# Supplementary material for: Knowledge of postpartum complications and associated factors among women who gave birth in the last 12 months in Arba Minch Town, Southern Ethiopia, 2019: A community-based cross-sectional study
Source: PLoS One. 2023 Feb 6;18(2):e0281242. doi: 10.1371/journal.pone.0281242 (PMC9901784; doi:10.1371/journal.pone.0281242)
Supplement: Attachment — Submitted filename: PONE-D-21-29865_R1.pdf [file pone.0281242.s005.pdf]

PLOS ONE

Knowledge of postpartum complications and associated factors among women gave birth in the last 12 months in Arba Minch Town, Southern Ethiopia, 2019: Community Based Cross-Sectional Study

--Manuscript Draft--

**Manuscript Number:**	PONE-D-21-29865R1
**Article Type:**	Research Article
**Full Title:**	Knowledge of postpartum complications and associated factors among women gave birth in the last 12 months in Arba Minch Town, Southern Ethiopia, 2019: Community Based Cross-Sectional Study
**Short Title:**	Knowledge of postpartum complications and associated factors among mothers who gave birth in last 12 months.
**Corresponding Author:**	Godana Tessema Yaya, MSC<br>Arba Minch University<br>Arba Minch, ETHIOPIA
**Keywords:**	Arba Minch town, complications, Cross sectional, Knowledge and Postpartum
**Abstract:**	ABSTRACT<br><br>Introduction : Knowledge of women about obstetric complications helps to seek health care earlier before obstetric complications arise. Most maternal deaths occur due to poor health seeking after childbirth, but little is done on maternal knowledge of postpartum complications. Therefore this study aimed to assess knowledge of postpartum complications and factors among women gave birth in the last 1year in Arba Minch Town, Ethiopia.<br>Methods: A community-based cross sectional study was conducted on 418 women from December 01 to 15, 2019. A multi-stage sampling method was applied to reach study units. A semi-structured questionnaire was used to collect the data using face to face interview. Bivariable and multivariable logistic regression was applied to examine the relationship between dependent and independent variables. Statistical significance was declared at a P-value < 0.05 with the corresponding 95% confidence level.<br>Results: Knowledge of women on postpartum complications was 23.9%. Secondary and above educational level (AOR = 3.82, 95% CI: [1.70, 8.65], Grand multiparity (AOR= 2.31, 95% CI: [1.13, 4.71], having four and above ANC visit (AOR = 2.04, 95% CI: [1.10, 3.81]) and self-decision making power to seek care (AOR = 3.68, 95% CI: [2.21, 6.11]) were statistically significant factors.<br>Conclusion and recommendation: Mother’s knowledge of postpartum complications was low in this study area. Empowering women educational level, decision making power to seek health care and counseling during ANC follow up will needed.
**Order of Authors:**	Godana Tessema Yaya, MSC
	Gistane Ayele, MSC,Ass. Proffessor
	Kassahun Fikadu, MSC
	Wanzahun Godana, MPH, Assistant professor, PHD Fellow
	Gebresilasea Gendisha, MSC, Assistant Professors
**Opposed Reviewers:**	
**Response to Reviewers:**	Dear editors and reviewers, Thank you for your helpful and constructive comments. I have a great appreciation for ideas you raised during review time. Responses to the specific reviewer and editor comments were fully explained in the rebuttal letter, which labeled as 'Response to the Reviewer' and uploaded separately.
**Additional Information:**	
**Question**	**Response**
**Financial Disclosure**	The author(s) received no specific funding for this work.

Powered by Editorial Manager® and ProduXion Manager® from Aries Systems Corporation

Enter a financial disclosure statement that describes the sources of funding for the work included in this submission. Review the submission guidelines for detailed requirements. View published research articles from *PLOS ONE* for specific examples.<br><br>This statement is required for submission and **will appear in the published article** if the submission is accepted. Please make sure it is accurate.<br><br>**Unfunded studies**<br>Enter: *The author(s) received no specific funding for this work.*<br><br>**Funded studies**<br>Enter a statement with the following details:<br>• Initials of the authors who received each award<br>• Grant numbers awarded to each author<br>• The full name of each funder<br>• URL of each funder website<br>• Did the sponsors or funders play any role in the study design, data collection and analysis, decision to publish, or preparation of the manuscript?<br>• **NO** - Include this sentence at the end of your statement: *The funders had no role in study design, data collection and analysis, decision to publish, or preparation of the manuscript.*<br>• **YES** - Specify the role(s) played.<br><br>* typeset	
**Competing Interests**<br><br>Use the instructions below to enter a competing interest statement for this submission. On behalf of all authors, disclose any competing interests that could be perceived to bias this work—acknowledging all financial support and any other relevant financial or non-financial competing interests.<br><br>This statement is required for submission and **will appear in the published article** if	The authors have declared that no competing interests exist.

Powered by Editorial Manager® and ProduXion Manager® from Aries Systems Corporation

the submission is accepted. Please make sure it is accurate and that any funding sources listed in your Funding Information later in the submission form are also declared in your Financial Disclosure statement.<br><br>View published research articles from *PLOS ONE* for specific examples.<br><br>**NO authors have competing interests**<br><br>Enter: *The authors have declared that no competing interests exist.*<br><br>**Authors with competing interests**<br><br>Enter competing interest details beginning with this statement:<br><br>*I have read the journal's policy and the authors of this manuscript have the following competing interests: [insert competing interests here]*<br><br>* typeset	
**Ethics Statement**<br><br>Enter an ethics statement for this submission. This statement is required if the study involved:<br><br>• Human participants<br>• Human specimens or tissue<br>• Vertebrate animals or cephalopods<br>• Vertebrate embryos or tissues<br>• Field research<br><br>Write "N/A" if the submission does not require an ethics statement.<br><br>General guidance is provided below. Consult the submission guidelines for detailed instructions. **Make sure that all information entered here is included in the Methods section of the manuscript.**	The letter of ethical clearance was obtained from Institutional Review Board (IRB) of College of Medicine & Health Sciences, Arba Minch University, Ethiopia.

Powered by Editorial Manager® and ProduXion Manager® from Aries Systems Corporation

**Format for specific study types**<br><br>**Human Subject Research (involving human participants and/or tissue)**<br>• Give the name of the institutional review board or ethics committee that approved the study<br>• Include the approval number and/or a statement indicating approval of this research<br>• Indicate the form of consent obtained (written/oral) or the reason that consent was not obtained (e.g. the data were analyzed anonymously)<br><br>**Animal Research (involving vertebrate animals, embryos or tissues)**<br>• Provide the name of the Institutional Animal Care and Use Committee (IACUC) or other relevant ethics board that reviewed the study protocol, and indicate whether they approved this research or granted a formal waiver of ethical approval<br>• Include an approval number if one was obtained<br>• If the study involved *non-human primates*, add *additional details* about animal welfare and steps taken to ameliorate suffering<br>• If anesthesia, euthanasia, or any kind of animal sacrifice is part of the study, include briefly which substances and/or methods were applied<br><br>**Field Research**<br><br>Include the following details if this study involves the collection of plant, animal, or other materials from a natural setting:<br>• Field permit number<br>• Name of the institution or relevant body that granted permission	
**Data Availability**<br><br>Authors are required to make all data underlying the findings described fully available, without restriction, and from the time of publication. PLOS allows rare exceptions to address legal and ethical concerns. See the PLOS Data Policy and FAQ for detailed information.	Yes - all data are fully available without restriction

A Data Availability Statement describing where the data can be found is required at submission. Your answers to this question constitute the Data Availability Statement and **will be published in the article**, if accepted.<br><br>**Important:** Stating 'data available on request from the author' is not sufficient. If your data are only available upon request, select 'No' for the first question and explain your exceptional situation in the text box.<br><br>Do the authors confirm that all data underlying the findings described in their manuscript are fully available without restriction?	
**Describe where the data may be found in full sentences. If you are copying our sample text, replace any instances of XXX with the appropriate details.**<br><br>• If the data are **held or will be held in a public repository**, include URLs, accession numbers or DOIs. If this information will only be available after acceptance, indicate this by ticking the box below. For example: *All XXX files are available from the XXX database (accession number(s) XXX, XXX.*).<br>• If the data are all contained **within the manuscript and/or Supporting Information files**, enter the following: *All relevant data are within the manuscript and its Supporting Information files.*<br>• If neither of these applies but you are able to provide **details of access elsewhere**, with or without limitations, please do so. For example:<br><br>*Data cannot be shared publicly because of [XXX]. Data are available from the XXX Institutional Data Access / Ethics Committee (contact via XXX) for researchers who meet the criteria for access to confidential data.*<br><br>*The data underlying the results presented in the study are available from (include the name of the third party*	All relevant data are within the manuscript and its supporting information files. But if there is additional data required, I can able to provide details of any required data elsewhere, without limitations.<br>@ htt://orcid.org/0000-0002-4673-6975

and contact information or URL).<br>• This text is appropriate if the data are owned by a third party and authors do not have permission to share the data.<br><br>* typeset	
Additional data availability information:	

Powered by Editorial Manager® and ProduXion Manager® from Aries Systems Corporation

Cover Letter

Dear Editors

We conducted research entitled "**Knowledge of postpartum complications and associated factors among women who gave birth in the last 12 months in Arba Minch Town, Southern Ethiopia, 2019: Community-Based Cross-Sectional Study.**"

As it is known types of researchseveral researches were conducted on maternal knowledge of obstetrics complications and associated factors. However, little is done on three separated phases of obstetrics i.e. pregnancy, childbirth, and postpartum periods. In this phase maternal health care as well as their knowledge on complications to this specific period is different. Therefore, this study focused on women's knowledge of postpartum complications and associated factors. Because in this obstetric phase most the mothers lost their life due to complications that happened to them.

So, we hope this is of a good stand to contribute to filling the gap. Therefore, we hope the article will be of your standard to be published.

Based on the reviewed comments the following ideas were revised to the submition form.

- Funding statement: 'Author received specific funding from Arba Minch University for this work.'
- Data availability statement: All needed data were within the document and for further by ORCID-Access at; http://orcid.org/0000-0002-4673-6975
- Ethical statement: Also written consent was taken from study participants during data collection …Should be removed. Further revisions were done to the manuscript

Godana Yaya

Corresponding author and on the behalf of the other co-authors

Corresponding author: Godana Yaya [1*](E-mail: godanayaya8@gmail.com or godana.yaya@amu.edu.et)

Co-authors: Gistane Ayele [2@](gistane.ayele@amu.edu.et), Kassahun Fikadu [1@](kassahun.fikadu@amu.edu.et), Wanzahun Godana [2@](wanzahun.godana@amu.edu.et),Gebresilasea Gendisha[1@](gebresilasea@yahoo.com)

[1] Department of Midwifery, College of Medicine and Health Sciences, Arba Minch University, Arba Minch, Ethiopia

[2] School of Public Health, College of Medicine and Health Sciences, Arba Minch University, Arba Minch, Ethiopia

Hint:

*Principal Author

@Coauthors authors & contributed equally to this work

Manuscript Click here to access/download;Manuscript;Manuscript.docx

Knowledge of postpartum complications and associated factors among women gave birth in the last 12 months in Arba Minch Town, Southern Ethiopia, 2019: Community-Based Cross-Sectional Study

Corresponding author: Godana Yaya [1*](E-mail: godanayaya8@gmail.com or godana.yaya@amu.edu.et)

Co-authors:

Gistane Ayele [2@](gistane.ayele@amu.edu.et),

Kassahun Fikadu[1@](kassahun.fikadu@amu.edu.et),

Wanzahun Godana[2@](wanzahun.godana@amu.edu.et),

Gebresilasea Gendisha[1@](gebresilasea@yahoo.com)

[1] Department of Midwifery, College of Medicine and Health Sciences, Arba Minch University, Arba Minch, Ethiopia

[2] School of Public Health, College of Medicine and Health Sciences, Arba Minch University, Arba Minch, Ethiopia

Hint:

* Corresponding author

@Co-authors: Contributed equally to this work

ABSTRACT

them

Introduction: Knowledge of women about obstetric complications helps to seek health care earlier before obstetric complications arise. Most maternal deaths occur due to poor health-seeking after childbirth, but little is done on maternal knowledge of postpartum complications. Therefore this study aimed to assess knowledge of postpartum complications and associated factors among women who gave birth in the last 12 months in Arba Minch Town, Sothern Ethiopia,2019: Community-Based cross-sectional Study

Methods: A community-based cross-sectional study was conducted on 418 women from December 01 to 15, 2019. A multi-stage sampling method was applied to reach study units. A semi-structured questionnaire was used to collect the data using face-to-face interviews. Bivariable and multivariable logistic regression was applied to examine the relationship between dependent and independent variables. Statistical significance was declared at a P-value $\leq$ 0.05 with the corresponding 95% confidence level.

Results: Knowledge of women on postpartum complications was 23.9%. Secondary and above educational level (AOR = 3.82, 95% CI: [1.70, 8.65]), Grand multiparity (AOR= 2.31, 95% CI: [1.13, 4.71]), having four and above ANC visit **(**AOR **=** 2.04, 95% CI: [1.10, 3.81]) and self-decision making power to seek care (AOR = 3.68, 95% CI: [2.21, 6.11]) were statistically significant factors.

Conclusion and recommendation: Mothers' knowledge of postpartum complications was low in this study area. Improving women's educational level, decision-making power to seek health care, and counseling during ANC follow-up may be useful approaches to increase their knowledge of postpartum complications.

INTRODUCTION

The postpartum period is a time that starts one hour after delivery and extends up to 42 complete days postpartum. It is a critical phase in the lives of mothers and newborns. During this time, a wide range of postpartum complications have been reported, including excessive or prolonged postpartum bleeding, breastfeeding problems, urinary incontinence, constipation, depression and psychoses, post-traumatic stress disorder, anxiety, fatigue, constipation, and sleep disorders.[1, 2]

Even though major changes and complications that threaten the lives of mothers and newborns occur during this period, there is a lack of appropriate care during this period, which results in maternal morbidity and mortality. The majority of maternal deaths occur within the first month of childbirth, with nearly half occurring within the first 24 hours and 66% occurring within the first week [3].

In 2015, around 303, 000 women died due to pregnancy and childbirth-related complications in the world. Almost all (99%) maternal deaths occur in developing countries, of which 66% of maternal deaths occur in Sub-Saharan African countries, which are estimated to have 546 maternal deaths per 100,000 live births. Most of these deaths could have been prevented if mothers had sought health care before complications worsened. The new transformative agenda set by the United Nations (UN) Sustainable Development Goal (SDG) is planned to reduce the global maternal mortality ratio (MMR) to less than 70 per 100,000 live births by 2030[4-6].

Among pregnancy and childbirth-related complications, hemorrhage was the leading direct cause of maternal death worldwide, representing 27.1% of maternal deaths. Postpartum hemorrhage alone accounted for 19.7% of total maternal deaths due to hemorrhage. Hypertension was the second most common direct cause worldwide. Maternal mortality due to sepsis was 10.7% and other deaths were due to other direct causes [7].

According to the Ethiopian Demographic Health Survey (EDHS), the estimated pregnancy-related mortality ratio is 412 deaths per 100,000 live births. In Ethiopia, for every 1,000 live births, approximately four women die during pregnancy, childbirth, or within 2 months after

childbirth. Although Ethiopia is one of the countries that makes progress toward achieving MDG 5, still Maternal Mortality Ratio (MMR) in the country is high [6, 8].

The postpartum period is associated with significant biosocial changes in women's lives. When these changes occur, they can be associated with problems such as vaginal bleeding foul-smelling vaginal discharge, constipation, tenderness and pain in the perineum, breast problems, burning during urination, wound breakdown, self-consciousness, and loneliness. If these problems progress, they can lead to complications that can negatively affect women's health and daily life after the postnatal period. Women's knowledge of these associated problems is deemed necessary to lower postpartum complications [9].

Many factors influence the outcome of pregnancy starting with the onset of any obstetric complications. The delayed treatment has the greatest negative impact on the outcome, which is due to a variety of factors, including a lack of information and adequate knowledge about complications. According to one study, 80 percent of maternal deaths in Ethiopia occurred at home [10].

"Delay I" is the leading cause of maternal mortality in Ethiopia, and the main cause of "delay I" is a lack of knowledge about pregnancy-related issues. Women's knowledge is important for seeking medical care before complications arise and has a crucial influence on the reduction of maternal morbidity and mortality [11].

Despite several types of research being conducted on maternal knowledge of obstetric complications and associated factors, little is done on three separated phases of obstetrics, i.e., pregnancy, childbirth, and postpartum periods. In this phase, maternal health care as well as their knowledge of complications specific to this specific period is different. Therefore, this study was focused on women's knowledge of postpartum complications and associated factors.

METHODS & MATERIAL

Study Area and Period

The study was conducted from December 1- 15/ 2019 in Arba Minch town. Arba Minch is the administrative town of Gamo Zone, South Nation Nationality People Region (SNNPR), and Ethiopia. It is found at an elevation of 1285 meters above sea level and is located 505 km away from Addis Ababa, the capital city of Ethiopia. People with different ethnic groups and religions reside in the town. The town is divided into four sub-cities, namely, Abaya, Sikela, Nech-Sar & Shecha. The town consists of 11 kebeles (the smallest administrative unit) with a total population of 112, 724. There are 26, 265 reproductive age group (15-49) women who reside in the town out of which 4428 were pregnant. The total number of mothers who gave birth at the facility preceding the study period was 3992. There is one governmental General Hospital, two Health Centers, 17 primary care clinics, and 14 medium-sized private clinics in the town [12].

Study design

A community-based cross-sectional study was conducted.

Population

As the source population, all women who gave birth in the last 12 months before the start of the study and lived in Arba Minch town for six months were chosen. Individual mothers were systematically selected and interviewed in four randomly selected kebeles, with a total of 1475 mothers eligible for the study sample allocation. Mothers who were critically ill and unable to participate in the interviews were excluded from the study. As a result, the study included those mothers who had given birth within the previous year before the study period.

Sample size determination and sampling procedures

Sample size determination

The sample size was calculated by using a single population proportion formula with the assumption of, a 95% confidence level, 5% margin of error, 5% non- respond rate, 1.5 design effect, and expected population proportion of mothers who were knowledgeable about postpartum complications was 22.1% [13].

$$n = \frac{\left(z_{\frac{\propto}{2}}\right)^2 . p(1-p)}{d^2}$$

Where,

n= sample size

Z= standard normal distribution value at 95% confidence level of $\frac{\propto}{2}$ = 1.96

P= women's knowledge of postpartum complications = 22.1%

d= margin of error= 5%

$$n = \frac{(1.96)^2 \times 22.1(1-0.221)}{0.05^2}$$

n = **<u>265</u>**

Based on the assumptions the final calculated sample was**: <u>n = 418</u>**

Sampling procedures

Arba Minch town was purposively selected as a study area. The study participants were chosen using a multistage sampling process. There were 11 kebeles in the town, so kebeles were divided into 11 clusters. Using a simple random sampling procedure, four kebeles (Bere, Ediget-Ber, Wuha-Minch, and Dilfana) were chosen, and mothers who gave birth in the previous 12 months in each kebele were identified by contacting kebele health extension workers (HEW) for the updated register. The overall number of mothers giving birth in the four kebeles was N (estimated total number of deliveries in four kebeles) 1475 (Bere = 331, Ediget-ber = 471, Wuha-Minch = 371 & Dilfana = 302 ladies). Using a proportional allocation formula, the sample assigned to a specific kebele was considered. The calculated samples for Bere, Ediget Bere, Wuha Minch, and Dilfana were 94, 133, 105, and 86 mothers, respectively, based on the proportion to size allocation. The 'K'-value was calculated by dividing the number of mothers who gave birth in the previous 12 months in each selected kebele by the sample allocated to that kebele, yielding the following results: 3.52, 3.54, 3.53, and 3.51 for Bere, Ediget Ber, Wuha Minch, and Dilfana, respectively. By chance, the rounded k-value of all of the chosen kebeles was 3. The first mother was discovered by randomly selecting one household on the kebele's outskirts with the assistance of HEW and the leaders of specific kebeles. Then, for every three mothers, a systematic random sampling

technique was used to obtain study participants, which was repeated until the sample was complete. If more than one mother was found in a single household using the lottery method, only one mother was chosen.

Data collection tools and procedures

The data were collected by face-to-face interviews using a semi-structured questionnaire. The questionnaire was prepared from previously approved kinds of literature considering the study objectives [17, 18]. It consists of socio-demographic variables, obstetric factors, and postpartum complications knowledge measuring questions. The questionnaire was prepared in English and translates to Amharic (working language of the study area) and then, back to English for consistency.

Three female diploma midwives who are fluent in speaking the Amharic language were involved in data collection. Two males who have Bachelors of Science degrees in midwifery' health professionals were recruited as supervisors.

Study variables

Dependent variable: Women's knowledge of postpartum complications

Independent variables

Socio-demographic variables: Maternal age**,** marital status**,** occupational status**,** educational status**,** monthly income **&** access to media

Obstetric factors & health service use: Parity**,** ANC visit**,** information on the pregnancy-related problem**,** place of delivery**,** self-decision making autonomy to seek health care

Operational definition

Knowledgeable: Mothers who spontaneously mentioned three and above postpartum complications were declared as knowledgeable about postpartum complications [13, 14].

Data quality management

The quality of the data was maintained before, during, and after data collection.

Before data collection, a semi-structured questionnaire was adapted from previously published kinds of literature. Pretesting of the questionnaire was carried out on 5% of the

sample that was, on 21 mothers in Mirab Abaya town, Gamo Zone, Ethiopia, and any necessary amendments were done.

Intensive training of three days duration about the objective, questionnaire, ethical issues, and pre-test of the questionnaire was given to data collectors and supervisors.

During the data collection period, questionnaires were checked for completeness and consistency immediately by data collectors

After data collection, the principal investigator and supervisors rechecked the collected data for its completeness, and corrective measures were taken accordingly. Then, data was entered to epi info version 3.5.1 and 5% of the data set was double entered to check the accuracy of the entered data

Data processing and analysis

After data collection was complete, each questionnaire was coded entered into Epi info version 3.5.1 and then, it was exported to SPSS Version 20 window compatible software for cleaning and analysis. By using simple frequency tables and cross-tabulation data were checked for completeness.

A descriptive statistical method such as frequency tables, graphs, and mean with standard deviation was used to present different characteristics of study participants.

Bivariable logistic regression was done and variables with P-value ≤ 0.25 and all other variables that had an association in previously approved kinds of literature and assumed to have scientific relevance to the study were selected as candidate variables for multivariable logistic regression analysis to control the effect of confounders. A backward stepwise logistic regression method was used for the analysis. Model fitness was checked by Hosmer and Lemeshow's goodness of model fit test (P=0.434).

Finally adjusted odds ratio with corresponding 95% CI and p-value ≤ 0.05 was taken as a statistical association between the dependent and independent variables.

Ethical consideration

An ethical clearance letter was obtained from the Institutional Research Review Board (IRRB) of the College of Medicine & Health Sciences (CMHS), Arba Minch University

(AMU). (See Figure1). A formal letter of permission to conduct the study was obtained from Gamo Zone Health Department, Arba Minch Town Health Office. The verbal consensus was made with mothers after an explanation of the purpose of the study, and the part they took in the research by data collectors to assure their right to refuse or participate in the study. Mothers were also told that the data obtained will be kept confidential & had the right to withdraw from the study at any time during the interview.

RESULTS

Socio-demographic characteristics of study participants

A total of 418 women who gave birth in the twelve months preceding the study period were recruited to participate and resulting in a 100 % response rate. The respondents' average age was 28.35 (SD 5.84) with a minimum and maximum of 18 and 42 ages respectively. The majority of respondents 202(48.3 %) and 181(43.3 %) were Orthodox and Protestant, respectively, and 400(95.7 %) were married. The majority of respondents, 267 (63.9 %), were Gamo ethnic group and 238 (56.9 %) were housewives. In terms of educational attainment, 222 (53.1 %) of respondents have completed Secondary School or higher. Among the total study participants, 367 (87.8 %) have access to mass media, with television being the most commonly used medium 335 (80.1 %) (**See Table 1.**)

Obstetric characteristics of mothers and health service utilization

Among the 418 mothers polled, 188 (45%) were multiparous, and 100 (23.9%) were grand multiparous. 411 (98.3 percent) of the total study participants attended ANC follow-ups for their most recent pregnancy. 283 (67.7 percent) of mothers who had a history of ANC visits had attended four or more times. The majority of the attendees had visited government facilities, with only 6 (1.4 percent) having visited a private clinic. Participants in the study were also asked whether or not health care providers provided information on pregnancy-related issues during visits to the ANC clinic. 275 (65.8 percent) of respondents received information about pregnancy-related problems from a health care provider, while 136 (32.5 percent) did not. The vast majority of mothers, 392 (93.8%), gave birth in a health facility, while 26 (6.2%) gave birth at home. Women were also asked if they were the primary decision-makers in their families when it came to seeking medical attention for pregnancy-related issues. Only 127 (30.4 percent) of total respondents were self-decision makers, and

291 (69.6 percent) of women's decisions were accompanied by their husbands and other family members. (**See Table 2**).

Knowledge of postpartum complications

Even though the majority of the study participants 260(62.2 percent) had heard about postpartum complications before the study, only 100 (23.9 percent) of 418 women had Knowledge of postpartum complications (knew three or more postpartum complications). The main sources of information mentioned by respondents were: health personnel during ANC follow up 180 (43.1 percent), HEW (health extension workers) discussions on maternal health 143 (34.2 percent), mass media 83 (19.9 percent), school 18 (4.3 percent), and previous history 9 percent (2.2 percent). Severe vaginal bleeding (240, or 57.4 percent) was the most frequently mentioned postpartum complication, followed by pregnancy-induced hypertension after childbirth (121, or 28.9 percent). Breast complications were mentioned 75 (17.9 percent), blurred vision and weakness were mentioned 73 (17.5%), foul-smelling vaginal discharge was mentioned 71 (17%), convulsions were mentioned 14 (3.3%), and postpartum psychoses and blues were mentioned the least 4 (1%). The majority of respondents who had heard about postpartum complications expressed a desire to visit health care facilities if complications arose. **(See Figure 2)**

Factors associated with Women's knowledge of postpartum complications

After multivariable logistic regression was done; some variables such as secondary and above educational status, grand multipara, four and above the frequency of ANC visit and decision making power of mothers to seek health care utilization were significantly associated with maternal knowledge of postpartum complications.

The educational status of the mothers showed statistical association with knowledge of postpartum complications, (AOR = 3.82, 95% CI: [1.70 – 8.65]).

Grand multipara mothers had also an association with the dependent variable, (AOR= 2.31, 95% CI: [1.13 – 4.71]). Similarly, frequency of antenatal care was another significant predictor of women’s knowledge of postpartum complications, **(**AOR = 2.04, 95% CI: [1.10 – 3.81]).

Another variable that was associated with maternal knowledge of postpartum complications was the decision-making power of mothers to seek health service utilization, (AOR = 3.68, 95% CI: [2.21, 6.11]). **(See Table 3).**

DISCUSSION

This study reported that 23.9% of the study participants were knowledgeable on postpartum complications.

This study finding is much higher than a similar study conducted in Western Bengal of the Indian state, in 2011, in which the percentage of mothers who were knowledgeable on postpartum complications was 1.1% [15]. This difference could be due to variation in socio-demographical and time duration of the study.

The finding of a cross-sectional study carried out in rural area Gurage Zone, Ethiopia that reported 20.3% of mothers had mentioned two and above complications after childbirth, but in this study, 23.9% of mothers had mentioned three and above postpartum complications [16]. This study discrepancy might be due to the difference of study areas.

This study finding is slightly similar to cross-sectional studies conducted in two districts of the country; the Goba and Raya Kobo districts of Ethiopia that showed 22.1% & 26.4% of the mothers were knowledgeable on postpartum complications respectively [13, 14]. This consistence might be due to the similarity of the studies; in declaring mothers who had mentioned three and above complications after childbirth as knowledgeable.

The study finding is also less as compared with studies conducted in different parts of Ethiopia, such as Aleta Wondo Sidama (37.7%), Fango district Wolita (55.8%), & Debre Birhan Amhara (77.2%) [17-19]. This difference might be mothers who could mention two and above complications were considered as knowledgeable. But in the current study mothers were declared as knowledgeable if they mentioned three and above complications after childbirth.

This study revealed that severe vaginal bleeding was the most commonly known postpartum complication mentioned by study participants followed by hypertension, breast complication & foul-smelling discharge. This result is slightly in line with different research findings

conducted in other countries as well as in Ethiopia [20-22]. The least mentioned complication was postpartum psychoses and blues which were never mentioned in other research findings. This might be due to maternal history of previous experience of the complication.

In this study; educational status, being grand multiparity, the number of ANC visits and the decision-making power of mothers to seek health care were predictors of maternal knowledge of postpartum complications.

The educational status of the mothers showed a statistical association with knowledge of postpartum complications. Women who attended secondary and above educational level were about 4 times (AOR = 3.82, 95% CI: [1.70, 8.65] more likely to be knowledgeable than mothers who didn't attend formal education. This was consistent with a research finding of Raya Kobo, Ethiopia [14].

Similarly, the frequency of antenatal care was another predictor of women's knowledge of postpartum complications. And women who had four and above ANC follow up were 2 times **(**AOR **=** 2.04, 95% CI: [1.10, 3.81]) more likely to be knowledgeable on postpartum complications than women who had three or fewer ANC visits. This study finding is similar to different studies conducted in Ethiopia [23, 24].

Being grand multiparity also has a relationship with maternal knowledge of postpartum complications. Grand multipara mothers were 2 times (AOR= 2.31, 95% CI: [1.13, 4.71]) more likely to have knowledge of complications of childbirth than primipara mothers. This is similar to the institutional-based cross-sectional (2014) study finding of Mechekel East Gojjam Zone, Ethiopia [25].

Another very important variable that was associated with maternal knowledge of postpartum complications, with a p-value of < 0.001 was the decision-making power of mothers to seek health service utilization. Mothers who decide health service utilization in their household had 4 times (AOR = 3.68, 95% CI: [2.21, 6.11]) a likelihood to be knowledgeable than mothers who depend on the decision of other family members.

This is in line with study findings conducted in Arba Minch town, Ethiopia; that test association for general knowledge of obstetrical complications of the three periods [24].

LIMITATION & STRENGTH OF THE STUDY

Due to the nature of the cross-sectional study design, this study couldn't establish cause and effect relationships. This is the main limitation of the study.

As a strength, the study was conducted at the community level, which increases the generalizability to the entire urban area.

CONCLUSION AND RECOMMENDATIONS

Conclusion

According to this result mother's knowledge of postpartum complications is low. The most known complication after childbirth was severe vaginal bleeding followed by hypertension. Educational status, being grand multipara, the number of ANC visits and the decision-making power of mothers to seek health care were statistically associated variables that will predict maternal knowledge of postpartum complications.

Recommendations

Improving women's educational status and decision-making power to seek health care before complications happened is needed. Increasing information delivery system about specific postpartum complications during antenatal follow-up is the recommended intervention. Based on the study findings the following concerned bodies should be recommended to improve and strengthen women's knowledge of postpartum complications.

- Health personnel and health extension workers should strengthen awareness creation activities by conducting maternal discussion forums at the community level.
- The Ministry of health should continue to encourage pregnant mothers to attend four and above antenatal visits.
- Health extension workers and community leaders should mobilize the society about the advantage of women's self decision-making power to seek health care at the household level.
- The Ministery of education Should continue to encourage women's education.
- Further research supported with qualitative data will be needed.

ACKNOWLEDGMENTS

Acknowledgment

We would like to acknowledge Arba Minch University College of Medicine and Health Sciences for providing with necessary financial & material and making the work successful. We are thankful to all the participants who invested their time and perspectives with us.

REFERENCES

1. Health, F.M.o., *Management protocol on selected obstetrics topics*. 2010, Federal Democratic Republic of Ethiopia.
2. Huang, K., et al., *Does delivery mode affect women's postpartum quality of life in rural China?* Journal of clinical nursing, 2012. **21**(11- 12): p. 1534-1543.
3. Organization, W.H., *WHO recommendations on postnatal care of the mother and newborn*. 2014: World Health Organization.
4. Alkema, L., et al., *Global, Regional, and National Levels and Trends in Maternal Mortality Between 1990 and 2015, With Scenario-based Projections to 2030.* Obstetric Anesthesia Digest, 2016. **36**(4).
5. Nations, U., *The Sustainable Development Goals Report 2016.* United Nations: New York, NY, USA, 2016.
6. Organization, W.H., *Trends in maternal mortality: 1990-2015: estimates from WHO, UNICEF, UNFPA, World Bank Group, and the United Nations Population Division: executive summary*. 2015, World Health Organization.
7. Say, L., et al., *Global causes of maternal death: a WHO systematic analysis.* The Lancet Global Health, 2014. **2**(6): p. e323-e333.
8. Demographic, Z.Z., *Health Survey: Key Indicators.* Rockville, Maryland, 2016.
9. Nazik, E. and G. Eryilmaz, *The prevention and reduction of postpartum complications: Orem's Model.* Nursing science quarterly, 2013. **26**(4): p. 360-364.
10. Abdella, A., *Maternal mortality trend in Ethiopia.* Ethiopian Journal of Health Development, 2010. **24**(1).
11. Berhan, Y. and A. Berhan, *Commentary: reasons for persistently high maternal and perinatal mortalities in Ethiopia: part III–a perspective of the "three delays" model.* Ethiopian Journal of health sciences, 2014. **24**: p. 137-148.
12. Office: A.M.t.H., *Report of the district health department;* 2019.
13. Bogale, D. and D. Markos, *Knowledge of obstetric danger signs among childbearing age women in Goba district, Ethiopia: a cross-sectional study.* BMC pregnancy and childbirth, 2015. **15**(1): p. 77.
14. Bililign, N. and T. Mulatu, *Knowledge of obstetric danger signs and associated factors among reproductive-age women in Raya Kobo district of Ethiopia: A community-based cross-sectional study.* BMC pregnancy and childbirth, 2017. **17**(1): p. 70.
15. Mukhopadhyay, D.K., et al., *Status of birth preparedness and complication readiness in Uttar Dinajpur District, West Bengal.* Indian Journal of public health, 2013. **57**(3): p. 147.

16. Zepre, K. and M. Kaba, *Birth preparedness and complication readiness among rural women of reproductive age in Abeshige district, Gurage zone, SNNPR, Ethiopia.* International journal of women's health, 2017. **9**: p. 11.
17. Hailu, M., A. Gebremariam, and F. Alemseged, *Knowledge about obstetric danger signs among pregnant women in Aleta Wondo District, Sidama Zone, Southern Ethiopia.* Ethiopian Journal of health sciences, 2010. **20**(1).
18. Gebre, M., A. Gebremariam, and T.A. Abebe, *Birth preparedness and complication readiness among pregnant women in Duguna Fango District, Wolayta Zone, Ethiopia.* PloS one, 2015. **10**(9).
19. Akibu, M., *Prevalence and Associated Factors of Postnatal Care Utilization.* 2016.
20. Echoka, E., et al., *Barriers to emergency obstetric care services: accounts of survivors of life-threatening obstetric complications in Malindi District, Kenya.* The Pan African medical journal, 2014. **17**(Suppl 1).
21. Ekabua, J.E., et al., *Awareness of birth preparedness and complication readiness in southeastern Nigeria.* ISRN obstetrics and gynecology, 2011. **2011**.
22. Karkee, R., et al., *The role of obstetric knowledge in utilization of delivery service in Nepal.* Health education research, 2014. **29**(6): p. 1041-1048.
23. Maseresha, N., K. Woldemichael, and L. Dube, *Knowledge of obstetric danger signs and associated factors among pregnant women in Erer district, Somali region, Ethiopia.* BMC women's health, 2016. **16**(1): p. 30.
24. Workineh, Y., et al., *Knowledge of obstetric danger signs and its associated factors in Arba Minch town, Ethiopia.* Am J Health Res, 2014. **2**(5): p. 255-9.
25. Amenu, G., et al., *Knowledge about danger signs of obstetric complications and associated factors among postnatal mothers of Mechekel District Health Centers, East Gojjam Zone, Northwest Ethiopia, 2014.* Scientifica, 2016. **2016**.

Figure

Click here to access/download;Figure;Figure 1 Ethical Clerance letter.jpg

አርባ ምንጭ ዩኒቨርሲቲ
ARBA MINCH UNIVERSITY
የሕክምናና ጤና ሣይንስ ኮሌጅ
College of Medicine & Health Sciences (CMHS)
ተቋማዊ የምርምር ስነ-ምግባር ግምገማ ቦርድ ቢሮ
Institutional Research Ethics Review Board (IRB) Office

ፖ.ሣ.ቁ. 21
P.O.Box
አርባምንጭ ኢትዮጵያ
Arbaminch, Ethiopia

ስልክ
Tel
+251-46-8814014
+251-46-1817742
+251-46-8810070
+251-46-8810534

ቁጥር IRB/132/12
Ref. No.

ቀን 27/11/2019
Date

To: Godana Yaya (Principal investigator)

Subject: Cooperation for Research work and Notifying Ethical Clearance

Study Title: "Knowledge on postpartum complication and associated factors among women who gave birth in the last twelve months in Arba Minch town, southern Ethiopia".

We have received the research proposal to be under taken in the title mentioned above. At its session, the Institution Review Board (IRB) of College of Medicine & Health Sciences (CMHS), Arba Minch University (AMU) on 27 November, 2019 has discussed over the issue.

We are pleased to inform you that the above research proposal has been approved by the Institution Review Board (IRB of CMHS/AMU) as ethically sound research for implementation with close supervision. Further audit will be made in due course whenever necessary. This ethical clearance issued is valid for six (6) months. Therefore, we declare you to carry out the research undertaking as per the document of the proposal submitted.

With Regards,

ማናዬ ይሁኔ ተሻለ
Manaye Yihune Teshale
Chairperson, Institutional Research Ethics Review Board

CC:

- ✓ College of Medicine and Health sciences
- ✓ CMHS-Research coordination office
- ✓ IRB-CMHS/AMU Office

A.M.U

E-mail: irb.amu@yahoo.com

መልስ ሲጽፉልን የኛን ቁጥር ይጥቀሱ
When replying please indicate our reference No.

+251-46-8810279
Fax: +251-46-8810820

Figure

Click here to access/download;Figure;Table 1 Sociodemographic.tif

Table 1: Sociodemographic characteristics of the respondents in Arba Minch town, Southern Ethiopia, December 2019 (n=418).

Variables		Frequency(n=418)	Percent (%)
**Age**	15 - 19 Years	18	4.3
	20 – 24 years	97	23.2
	25 - 29 years	136	32.5
	≥ 30 years	167	40
**Religion**	Orthodox	202	48.3
	Protestant	181	43.3
	Muslim	35	8.4
**Marital status**	Married	400	95.7
	Others*	18	4.3
**Ethnicity**	Gamo	267	63.9
	Amhara	35	8.4
	Konso	31	7.4
	Derashe	24	5.7
	Gofa	21	5
	Zeyissie	18	4.3
	Others**	22	5.3
**Occupation**	Gov't employee	74	17.7
	Merchant	80	19.1
	Housewife	238	56.9
	Other**	26	6.2
**Income**	< 500 ETB	278	66.5
	500 – 1000 ETB	43	10.3
	1001 – 1500 ETB	18	4.3
	≥ 1501 ETB	79	18.9
**Women's educational**	Not attend formal education	96	23
	Primary	100	23.9
	Secondary &above	222	53.1
**Have media**	Yes	367	87.8
**Medias used by mothers**	Television	335	80.1
	Radio	148	35.4
	Newspaper	16	3.8

*include Widowed, Divorced **Gurage, Oromo, Wolaita, Silte, & Kore, **Student

Figure

Click here to access/download;Figure;Table 2 Obstetric characterstics.tif

Table 2: Obstetric characteristics and health service utilization of the respondents in Arba Minch town, Southern Ethiopia, December 2019.

Variables		Frequency(n=418)	Percent(%)
**Parity**	Primiparous	130	31.1
	Multiparous	188	45
	Grand Multipara	100	23.9
**Have ANC visit**	Yes	411	98.3
	No	7	1.7
**Frequency of ANC visit**	≥ 4 visit	283	67.7
	1 – 3 visit	128	30.6
**Institution of ANC visit**	Hospital	221	52.9
	Health center	192	45.9
	Private clinic	6	1.4
**Informed on pregnancy-related problem**	Yes	275	65.8
	No	136	32.5
**Place of delivery**	Health institution	392	93.8
	Home	26	6.2
**Decide to seek health care**	Self	127	30.4
	Husband/Family	291	69.6

Figure

Click here to access/download;Figure;Figure 2 Postpartum complications.tif

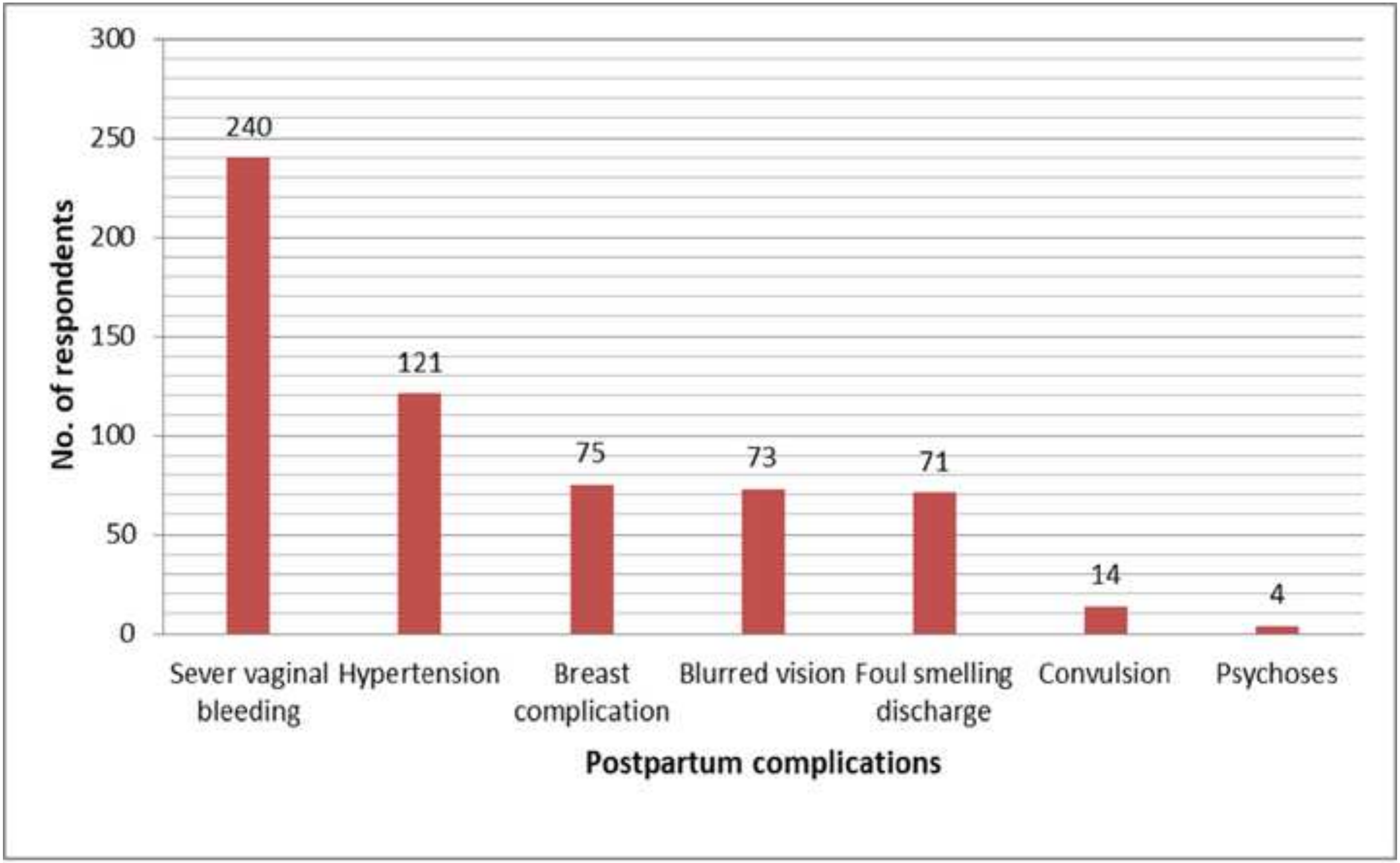

Figure 2: Postpartum complications mentioned by women who gave birth in the last 12 months in Arba Minch town, Southern Ethiopia, December 2019.

Table 3: Factors associated with mothers' knowledge of postpartum complications in Arba Minch town, Southern Ethiopia, 2019 (n = 418).

Variables	Categories	Knowledge of postpartum complications		COR (95% CI)	AOR(95%CI)
		Yes	No		
Age	15 - 19 Years	2	16	1	***
	20 – 24 years	16	81	1.58(0.33 - 7.56)	
	25 - 29 years	35	101	2.32(0.61 – 8.82)	
	≥ 30 years	47	120	3.13(0.69 – 14.16)	
Occupation	Gov't employee	27	47	1.92(0.69 – 5.35)	***
	Merchant	20	60	1.11(0.39 – 3.15)	
	Housewife	47	191	0.82(0.31 – 2.16)	
	Other****	6	20	1	
Income	< 500 ETB	56	222	1	***
	500 – 1000 ETB	10	33	1.20(0.56 – 2.58)	
	1001 – 1500 ETB	5	13	1.53(0.52 – 4.46)	
	≥ 1501 ETB	29	50	2.30(1.34 – 4.00)	
Educational status	Not attend formal education	9	87	0.20(0.10 – 0.43)	**3.82(1.70 – 8.65)***
	Attend primary school	16	84	0.37(0.20 – 0.68)	2.12(1.10 – 4.01)
	Secondary school and above	75	147	1	1
Access for media	Yes	95	272	3.21(1.24 - 8.33)	***
	No	5	46	1	
Parity	Primpara	25	105	0.64(0.35 – 1.20)	**2.31(1.13 – 4.71)**
	Multipara	48	140	0.93(0.54 – 1.61)	***
	Grand multipara	27	73	1	**1**
ANC visit	Yes	99	312	1.69(0.23 – 10.43)	***
	No	1	6	1	
Frequency of ANC	≥ 4	82	201	1	**1**
	1 – 3	17	111	0.38(0.21 – 0.67)	**2.04(1.10 – 3.81)****
Place of birth	Health institution	98	294	4.00(0.93 – 17.23)	***
	Home	2	24	1	
Decision making power	1= self	54	73	1	**1**
	2= family and husband	46	245	0.25(0.16 – 0.41)	**3.68(2.21 - 6.11)***

*Statistically significant variable at p-value < 0.01; ** significant variable at p-value <0.05; ***Variables which are not statistically significant in multivariable logistic regression ****Students

Supporting Information

Click here to access/download
Supporting Information
English & Amharic version questionnaire.docx

Knowledge of postpartum complications and associated factors among women gave birth in the last 12 months in Arba Minch Town, Southern Ethiopia, 2019: ~~Community Based~~ Community-Based Cross-Sectional Study

Corresponding author: Godana Yaya [1*](E-mail: godanayaya8@gmail.com or godana.yaya@amu.edu.et)

Co-authors:

Gistane Ayele [2@](gistane.ayele@amu.edu.et),

Kassahun Fikadu[1@](kassahun.fikadu@amu.edu.et),

Wanzahun Godana[2@](wanzahun.godana@amu.edu.et),

Gebresilasea Gendisha[1@](gebresilasea@yahoo.com)

[1] Department of Midwifery, College of Medicine and Health Sciences, Arba Minch University, Arba Minch, Ethiopia

[2] School of Public Health, College of Medicine and Health Sciences, Arba Minch University, Arba Minch, Ethiopia

Hint:

* Corresponding author

@Co-authors: ~~authors & contributed~~Contributed equally to this work

ABSTRACT

Introduction: Knowledge of women about obstetric complications helps to seek health care earlier before obstetric complications arise. Most maternal deaths occur due to poor ~~health seeking~~ health-seeking after childbirth, but little is done on maternal knowledge of postpartum complications. ~~Therefore~~ Therefore, this study aimed to assess knowledge of postpartum complications and factors among women gave birth in the last ~~1year~~ 12 Months in Arba Minch Town, Ethiopia.

Methods: A community-based ~~cross -sectional~~ cross-sectional study was conducted on 418 women from December 01 to 15, 2019. A multi-stage sampling method was applied to reach study units. A semi-structured questionnaire was used to collect the data using ~~face to face~~face-to-face ~~interview~~interviews. ~~Bivariable~~ Bi-variable and multivariable logistic regressions ~~was~~ were applied to examine the relationship between dependent and independent variables. Statistical significance was declared at a ~~P-value < 0.05~~ p-value ≤ 0.05 with the corresponding 95% confidence level.

Results: Knowledge of women on postpartum complications was 23.9%. Secondary and above educational level (AOR = 3.82, 95% CI: [1.70, 8.65]), Grand multi-parity (AOR= 2.31, 95% CI: [1.13, 4.71]), having four and above ANC visit (AOR = 2.04, 95% CI: [1.10, 3.81]) and self-decision making power to seek care (AOR = 3.68, 95% CI: [2.21, 6.11]) were statistically significant factors.

Conclusion and recommendation: Mother's knowledge of postpartum complications was low in this study area. ~~Empowering~~ Improving women's educational level, ~~decision making~~ decision-making power to seek health care, and counseling during ANC follow-up ~~will needed~~ may be useful approaches to increasing their knowledge of postpartum complications..

INTRODUCTION

The postpartum period is a time period that starts one hour after delivery and extends up to 42 complete days postpartum. It is a critical phase in the lives of mothers and newborns. During this time, a wide range of postpartum complications have been reported, including excessive or prolonged postpartum bleeding, breastfeeding problems, urinary incontinence, constipation, depression and psychoses, post-traumatic stress disorder, anxiety, fatigue, constipation, and sleep disorders.

The postpartum period is a time start one hour after delivery and extends up to 42 complete days of postpartum. It is a critical phase in the lives of mothers and newborns. A wide range of postpartum complications such as physical, mental, and emotional problems including excessive or prolonged postpartum bleeding, breast feeding problems, urinary incontinence, constipation, depression and psychoses, post traumatic stress disorder, anxiety, fatigue, constipation and sleep disorders have been reported during this period [1, 2]

Even though major changes and complications that threaten the lives of mothers and newborns occur during this period, there is a lack of appropriate care during this period, which results in maternal morbidity and mortality. The majority of maternal deaths occur within the first month of childbirth, with nearly half occurring within the first 24 hours and 66% occurring within the first week. Even though major changes and complications that threatening life of mothers and newborns occur during this period; there is a lack of appropriate care during this period which result in maternal morbidity and mortality. Most maternal deaths occur in the first month after child birth: almost half of postnatal maternal death occur within the first 24 hours and 66% occur during the first week [3].

In 2015, around 303, 000 women died due to pregnancy and childbirth-related complications in the world. Almost all (99%) maternal deaths occur in developing countries, of which 66% of maternal deaths occur in Sub-Saharan African countries, which are estimated to have 546 maternal deaths per 100,000 live births. Most of these deaths could have been prevented if mothers had sought health care before complications worsened. The new transformative agenda set by the United Nations (UN) Sustainable Development Goal (SDG) is planned to reduce the global maternal mortality ratio (MMR) to less than 70 per 100,000 live births by

2030In 2015, around 303, 000 women died due to pregnancy and childbirth related complications in the World. Almost all (99%) maternal deaths occur in developing countries, from which 66% of maternal death occurs in Sub-Saharan African countries which estimated for 546 maternal deaths per 100,000 live births. Most of these deaths could have been prevented if mothers seek health care before complications worsen. The new transformative agenda set by United Nations' (UN) Sustainable Development Goal (SDG) planned to reduce the global maternal mortality ratio (MMR) to less than 70 per 100,000 live births by 2030 [4-6].

Among pregnancy and childbirth related childbirth-related complications complications, hemorrhage was the leading direct cause of maternal death worldwide, representing 27.1% of maternal deaths. Postpartum hemorrhage alone accounted for 19.7% of total maternal deaths due to hemorrhage. Hypertension was the second most common direct cause worldwide. Maternal mortality due to sepsis was 10.7% and other deaths were due to other direct causes [7].

According to the Ethiopian Demographic Health Survey (EDHS), the estimated pregnancy-related mortality ratio is 412 deaths per 100,000 live births. In Ethiopia Ethiopia, for every 1,000 live births, approximately four women died during pregnancy, childbirth, or within 2 months after childbirth. Although Ethiopia is one among of the countries that makes a progress progress towards achieving MDG 5, still Maternal Mortality Ratio (MMR) in the country is high [6, 8].

The postpartum period is associated with significant biosocial changes in women's lives. When these changes occur, they can be associated with problems such as vaginal bleeding foul-smelling vaginal discharge, constipation, tenderness and pain in the perineum, breast problems, burning during urination, wound breakdown, self-consciousness, and loneliness. If these problems progress, they can lead to complications that can negatively affect women's health and daily life after the postnatal period. Women's knowledge on of these associated problems deemed is deemed necessary to lower postpartum complications [9].

There are many factors that affect Many factors influence the outcome of a pregnancy starting with the onset of any obstetric complications. The delayed treatment has the greatest negative impact on the outcome, which is due to a variety of factors, including a lack of

information and adequate knowledge about complications. According to one study, 80 percent of maternal deaths in Ethiopia occurred at home~~ the outcome of pregnancy from the onset of any obstetric complication. The outcome is most adversely affected by delayed treatment which is due to many factors and lack of information and adequate knowledge about complications. In Ethiopia, Study showed that, 80% of the maternal deaths took place at home ~~[10].

"Delay I" is the leading cause of maternal mortality in Ethiopia, and the main cause of "delay I" is a lack of knowledge about pregnancy-related issues.Women's knowledge is important for seeking medical care before complications arise and has a crucial influence on the reduction of maternal morbidity and mortality ~~'Delay I' is the highest contributing factor of maternal mortality in Ethiopia and lack of knowledge on pregnancy related problem is the main issue result in 'delay I. Women knowledge is important for seeking medical care before complications arise and has a crucial influence on reduction of maternal morbidity and mortality ~~[11].

Despite ~~of ~~several ~~researches ~~types of research ~~were ~~being conducted on maternal knowledge of obstetric~~s~~ complications and associated factors, little is done on three separated phases of obstetrics, i.e., pregnancy, child~~-~~birth, and postpartum periods. In this phase,~~s~~ maternal health care as well as their knowledge on complications to this specific period is different. Therefore, this study was focused on women's knowledge of postpartum complications and associated factors.

METHODS & MATERIAL

Study ~~area~~ Area and Period

The study was conducted from December 1- 15/ 2019 in Arba Minch town. Arba Minch is the administrative town of Gamo Zone, South Nation Nationality People Region (SNNPR), and Ethiopia. It is found at an elevation of 1285 meters above sea level and is located 505 km away from Addis Ababa, the capital city of Ethiopia. People with different ethnic groups and religions reside in the town. ~~are living in the town and they are followers of Orthodox Christianity, Protestants, Catholic and Muslim religions.~~ The town is divided ~~in to~~ into four ~~sub city such as;~~ sub-cities, namely, Abaya, Sikela, Nech-Sar & Shecha. ~~sub cities.~~ The town consists of 11 kebeles (the smallest administrative unit) with a total population of 112, 724. There are 26, 265 reproductive age group (15-49) women who reside in the town ~~among that~~ out of which 4428 were pregnant. The total number of mothers who gave birth at facility preceding the study period was 3992.~~The number of facility childbirth was 3992.~~ There is one governmental General Hospital, two Health Centers, 17 primary care clinics, and 14 medium-sized private clinics in the town ~~There is one governmental General Hospital, two Health Centers and 17 primary, 14 medium private clinics in the town~~ [12].

Formatted: Indent: Left: 0.5"

Study design

A community-based ~~cross~~ cross-sectional study was conducted.

Population

All women who gave birth in the last ~~1 year~~ 12 months preceding the date of commencement of the study and resided in Arba Minch town for six months were taken as source population. From four randomly selected Kebeles with 1475 total number of mothers eligible for the study sample allocation were done, and~~,~~ individual mothers were ~~selected by~~ systematically selected and interviewed. Mothers who were severely ill and unable to respond to the interviews were excluded from the study.

~~**Eligibility criteria**~~

~~**Inclusion criteria**~~

- Mothers who gave birth in the last 1 year Months preceding the study period.

- Who live in Arba Minch town for at least six Months?

Exclusion criteria

- Mothers those are severely ill and unable to respond.

Sample size determination and Sampling sampling procedures

Sample size determination

By Sample size was calculated by using a single population proportion formula with the assumption of, a 95% confidence level, 5% margin of error, 5% non- respond rate, 1.5 design effect to increase the chance of participation, and expected population proportion of mothers who were knowledgeable about postpartum complications were was 22.1%. The required sample size was 418 [13].

$$n = \frac{\left(z_{\frac{\alpha}{2}}\right)^2 . p(1-p)}{d^2}$$

Where,

n= sample size

Z= standard normal distribution value at 95% confidence level of $\frac{\alpha}{2}$= 1.96

P= women who have knowledge regarding postpartum complication = 22.1%

d= margin of error= 5%

$$n = \frac{(1.96)^2 \times 22.1(1-0.221)}{0.05^2}$$

n = 265

Based on the assumptions the final calculated sample was: **n = 418**

Formatted: Font: (Default) Times New Roman, 12 pt

Formatted: Normal, Indent: Left: 0.25", No bullets or numbering

Formatted: Font: (Default) Times New Roman, 12 pt

Formatted: Normal, Indent: Left: 0.75", No bullets or numbering

Sampling procedures

Arba Minch town was selected as a study area. A multistage sampling technique was applied to select study participants. There were a total of 11 Kebeles in the town and thus Kebeles were taken as 11 clusters. Among the Kebeles four Kebeles (Bere, Ediget-Ber, Wuha-Minch & Dilfana) were selected by simple random sampling method as a starting sampling technique. Firstly the number of, and mothers who gave birth in the last 12 months in each selected Kebeles was determined by contacting kebele health extension workers (HEW) for the updated registry. Then the total number of mothers who gave birth in the selected kebeles was 1475 (Bere = 331, Ediget-ber = 471, Wuha-Minch = 371 & Dilfana = 302 mothers) mothers. Using proportional allocation formula:- $n_i = \frac{n}{N}.N_i$

Where; n_i = Sample allocated to selected kebele (Bere, Ediget-ber, Wuha-minch & Dilfana)

N_i = Total number of mothers gave birth in each selected kebeles (Bere = 331, Ediget-ber = 471, Wuha-minch = 371 & Dilfana = 302)

$$n = Estimated\ total\ sample\ size\ of\ the\ study$$

N = Estimated total number of deliveries in four selected kebeles

Then According to the proportion to size allocation, the calculated sample for Bere, Ediget Bere, Wuha Minch and Dilfana were, 94, 133, 105, and 86 mothers respectively to each selected Kebeles was done. 'K'- value = 3 was determined by dividing a the number of mothers who gave birth in the last 12 months in each selected kebele by a sample allocated to the specific Kebele that, gave as, 3.52, 3.54, 3.53, and 3.51 for Bere, Ediget Ber, Wuha Minch, and Dilfana respectively. By chance, the rounded k-value of all the selected kebeles became 3. The first mother was found by randomly selecting one house hold household at the periphery of the kebeles by with the help of HEW and leaders of specific kebeles. Then, a Systematic random sampling technique was used every 3 mothers to obtain study participants and continued till the sample was fulfilled. If more than one mothers was encountered in one house hold household by using the lottery method one mother was selected.

Data collection tools and procedures

The data ~~was~~ were collected by face~~-to-~~-to-face interviews using a semi-structured questionnaire. The questionnaire was prepared from previously approved ~~literatures~~ kinds of literature considering the study objectives [17, 18]. It consists of ~~socio~~ socio-demographic variables, obstetric factors, and postpartum complications knowledge measuring questions. The questionnaire was prepared in English and translates to Amharic (working language of the study area) and then, back to English for consistency.

Three female diploma midwives who are fluent in speaking the Amharic language were involved in data collection. Two males who have Bachelors of Science degrees in midwifery' health professionals were recruited as supervisors.

Study variables

Dependent variable: Women's knowledge of postpartum complications

Independent variables

Socio-demographic variables: Maternal age**,** marital status**,** occupational status**,** educational status**,** monthly income **&** access ~~of~~ to media

Formatted: Justified

Obstetric factors & health service use: Parity**,** ANC visit**,** information on ~~pregnancy~~ the pregnancy-related problem**,** place of delivery**,** self-decision making autonomy to seek health care

Operational definition

Knowledgeable: Mothers who spontaneously mentioned three and above postpartum complications were declared ~~to have knowledge~~ as knowledgeable about postpartum complications [13, 14].

Data quality management

~~Quality~~ The quality of the data was maintained before, during, and after data collection.

Before data collection, a semi-structured questionnaire was adapted from previously published ~~literatures~~ kinds of literature. Pretesting of the questionnaire was carried out on 5% ~~(21)~~ of the sample that was, on 21 mothers in Mirab Abaya town, Gamo Zone, Ethiopia, and any necessary amendments were done.

Intensive training of three days duration about the objective, questionnaire, ethical issues, and pre-test of the questionnaire was given to data collectors and supervisors.

During the data collection period, questionnaires ~~was~~ were checked for completeness and ~~consistence~~ consistency immediately by data collectors

After data collection, the principal investigator and supervisors rechecked the collected data for its completeness, and corrective measures were taken accordingly. Then, data was entered to epi info version 3.5.1 and 5% of the data set was double entered to check the accuracy of the entered data

Data processing and analysis

After data collection was complete, each questionnaire was coded entered into Epi info version 3.5.1 and then, it was exported to SPSS Version 20 window compatible software for cleaning and analysis. By using simple frequency tables and ~~cross~~ cross-tabulation data were ~~cheeked~~ checked for completeness.

~~Descriptive~~ A descriptive statistical method such as frequency tables, graphs, and mean with standard deviation was used to present different characteristics of study participants.

Bivariable logistic regression was done and variables with P-~~-~~value ≤ 0.25 and all other variables that had an association in previously approved ~~literatures~~ kinds of literature were selected as candidate variables for multivariable logistic regression analysis to control the effect of confounders. ~~Backward~~ A backward stepwise logistic regression method was used for the analysis. Model fitness was checked by Hosmer and Lemeshow's goodness of model fit test.

Finally adjusted odds ratio with corresponding 95% CI and p-value ≤~~<~~0.05 was taken as a statistical~~ly~~ association between the dependent and independent variables.

Ethical consideration

~~Ethical~~ An ethical clearance letter was obtained from the Institutional Research Review Board (IRRB) of the College of Medicine & Health Sciences (CMHS), Arba Minch University (AMU). A formal letter of permission to conduct the study was obtained from Gamo Zone Health Department, Arba Minch Town Health Office. Verbal informed consent

was obtained from mothers after an explanation of the purpose of the study, and the part they took in the research by data collectors to assure their right to refuse or participate in the study. Mothers were also told that the data obtained will be kept confidential & had the right to withdraw from the study at any time during the interview.

RESULTS

Socio-demographic characteristics of study participants

A total of 418 women who gave birth in the twelve months preceding the study period were recruited to participate, and resulting in a 100 % respond rate. The respondents' average age was 28.35 (SD 5.84) with a minimum and maximum of 18 and 42 age respectively. The majority of respondents 202(48.3 %) and 181(43.3 %) were Orthodox and Protestant, respectively, and 400(95.7 %) were married. The majority of respondents, 267 (63.9 %), were Gamo ethnic group and 238 (56.9 %) were housewives. In terms of educational attainment, 222 (53.1 %) of respondents have completed Secondary School or higher. Among the total study participants, 367 (87.8 %) have access to mass media, with television being the most commonly used medium 335 (80.1 %) (**Table 1.**)

~~A total of 418 women who gave birth in the last twelve months preceding the study period were designed to participate in the study, this gives 100% response rate. The mean age of the respondents was 28.35 (SD ± 5.84). Most of the respondents were followers of Orthodox and Protestant religion, i.e. 202(48.3%) & 181(43.3%) respectively and 400(95.7%) of the study participants were married. The majority, 267(63.9%) of the respondents were Gamo ethnic group & 238(56.9%) were housewives. Regarding educational status of the respondents 222(53.1%) have attend Secondary School and above level. Among the total study participants 367(87.8%) have access of mass media and the commonly used Media was television, 335(80.1%) **(Table 1.)**~~

~~**Table 1: Socio-demographic characteristics of the respondents in Arba Minch town, Southern Ethiopia, December 2019 (n=418).**~~

~~Variables~~		~~Frequency(n=418)~~	~~Percent (%)~~
~~**Age**~~	~~15 - 19 Years~~	~~18~~	~~4.3~~
	~~20 – 24 years~~	~~97~~	~~23.2~~
	~~25 - 29 years~~	~~136~~	~~32.5~~
	~~≥ 30 years~~	~~167~~	~~40~~

~~**Religion**~~	~~Orthodox~~	~~202~~	~~48.3~~
	~~Protestant~~	~~181~~	~~43.3~~
	~~Muslim~~	~~35~~	~~8.4~~
~~**Marital status**~~	~~Married~~	~~400~~	~~95.7~~
	~~Others*~~	~~18~~	~~4.3~~
~~**Ethnicity**~~	~~Gamo~~	~~267~~	~~63.9~~
	~~Amhara~~	~~35~~	~~8.4~~
	~~Konso~~	~~31~~	~~7.4~~
	~~Derashe~~	~~24~~	~~5.7~~
	~~Gofa~~	~~21~~	~~5~~
	~~Zeyissie~~	~~18~~	~~4.3~~
	~~Others**~~	~~22~~	~~5.3~~
~~**Occupation**~~	~~Gov't employee~~	~~74~~	~~17.7~~
	~~Merchant~~	~~80~~	~~19.1~~
	~~Housewife~~	~~238~~	~~56.9~~
	~~Other**~~	~~26~~	~~6.2~~
~~**Income**~~	~~< 500 ETB~~	~~278~~	~~66.5~~
	~~500 – 1000 ETB~~	~~43~~	~~10.3~~
	~~1001 – 1500 ETB~~	~~18~~	~~4.3~~
	~~≥ 1501 ETB~~	~~79~~	~~18.9~~
~~**Women's educational**~~	~~Not attend formal education~~	~~96~~	~~23~~
	~~Primary~~	~~100~~	~~23.9~~
	~~Secondary &above~~	~~222~~	~~53.1~~
~~**Have media**~~	~~Yes~~	~~367~~	~~87.8~~
~~**Medias used by mothers**~~	~~Television~~	~~335~~	~~80.1~~
	~~Radio~~	~~148~~	~~35.4~~
	~~Newspaper~~	~~16~~	~~3.8~~

~~*include Widowed, Divorced **Gurage, Oromo, Wolaita, Silte, & Kore, **Student~~

Obstetric characteristics of mothers and health service utilization

Among 418 mothers 188(45%) were multipara and 100(23.9%) of the respondents were grand multiparous. From total study participants, 411(98.3%) respondents were attend ANC ~~follow~~ follow-ups for their last pregnancy. Among mothers who had a history of ANC visits 283(67.7%) of them had attended four and above times. Most of the attendants were visited government institutions and only 6(1.4%) attended ~~in~~ a private clinic. Study participants also asked whether health care providers delivered information on ~~pregnancy~~ pregnancy-related

problems or not during visiting the ANC clinic. From the respondents, 275(65.8%) were got information about pregnancy relate problems from a health care provider and 136(32.5%) were not. Most of the mothers 392(93.8%) were given their last birth at a health institution and 26(6.2%) at home. Women were also asked whether they were the primary ~~decision~~ decision-maker in their family regarding health seeking when ~~pregnancy~~ pregnancy-related problems occurred. Among total respondents, only 127(30.4%) women were self-decision makers, and 291 (69.6%) women's decisions were accompanied by their husbands and other family members. (Table. 2).

~~**Table 2: Obstetric characteristics and health service utilization of the respondents in Arba Minch town, Southern Ethiopia, December 2019.**~~

Variables		Frequency(n=418)	Percent(%)
Parity	Primiparous	130	31.1
	Multiparous	188	45
	Grand Multipara	100	23.9
Have ANC visit	Yes	411	98.3
	No	7	1.7
Frequency of ANC visit	≥ 4 visit	283	67.7
	1 – 3 visit	128	30.6
Institution of ANC visit	Hospital	221	52.9
	Health center	192	45.9
	Private clinic	6	1.4
Informed on pregnancy related problem	Yes	275	65.8
	No	136	32.5
Place of delivery	Health institution	392	93.8
	Home	26	6.2

~~Decide to seek health care~~	~~Self~~	~~127~~	~~30.4~~
	~~Husband/Family~~	~~291~~	~~69.6~~

Knowledge ~~on~~ of postpartum complications

From 418 total study participants, only 100(23.9%) women were knowledgeable on postpartum complications (knew three and above postpartum complications).

~~Majority~~ The majority of the study participants 260(62.2%) had information about postpartum complications. The main source of information mentioned by respondents ~~were~~ was, from health personnel during ANC follow up 180(43.1%), from discussion conducted on maternal health by HEW (health extension workers) 143(34.2%), mass media 83(19.9%) school 18(4.3%) and from previous history 9(2.2%).

The most frequently mentioned postpartum complication was severe vaginal bleeding 240(57.4%), followed by ~~pregnancy~~ pregnancy-induced hypertension after ~~child birth~~ childbirth 121(28.9%). The other mentioned complications were breast complication 75(17.9%), blurred vision and weakness 73(17.5%), ~~foul~~ foul-smelling vaginal discharge 71(17%), convulsions 14(3.3%) and the least mentioned was postpartum psychoses and blues. Most of the respondents that had information on postpartum complications have the interest to visit health institutions if thus complications occurred.

The main reason mentioned by mothers who had no ~~any~~ information about complications that could happen after ~~child birth~~ childbirth was never heard about any complications. (Figure. 1).

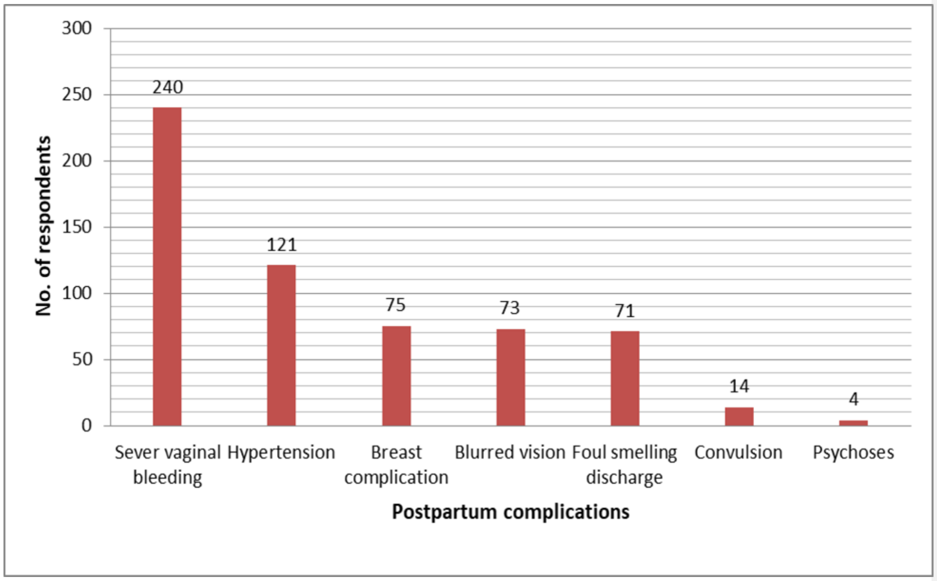

Figure 1: **Postpartum complications mentioned by women who gave birth in the last 12 months in Arba Minch town, Southern Ethiopia, December 2019.**

Factors associated with Women's knowledge of postpartum complications

After multivariable logistic regression was done; some variables such as secondary and above educational status, grand multipara, four and above the frequency of ANC visit and decision making power of mothers to seek health care utilization were significantly associated with maternal knowledge of postpartum complications.

~~Educational~~ The educational status of the mothers showed statistical association with knowledge of postpartum complications, (AOR = 3.82, 95% CI: [1.70 – 8.65]).

Grand multipara mothers had also an association with the dependent variable, (AOR= 2.31, 95% CI: [1.13 – 4.71]). Similarly, frequency of antenatal care was another significant~~ly~~ predictor of women's knowledge of postpartum complications, **(**AOR = 2.04, 95% CI: [1.10 – 3.81]).

Another variable that was associated with maternal knowledge of postpartum complications was the decision decision-making power of mothers to seek health service utilization, (AOR = 3.68, 95% CI: [2.21, 6.11]).(See Table 3.)

Table 3: Factors associated with mothers' knowledge of postpartum complications in Arba Minch town, Southern Ethiopia, 2019 (n = 418).

Variables	Categories	Knowledge of postpartum complications		COR (95% CI)	AOR(95%CI)
		Yes	No		
	15 – 19 Years	2	16	1	
	20 – 24 years	16	81	1.58(0.33 – 7.56)	
Age	25 – 29 years	35	101	2.32(0.61 – 8.82)	***
	≥ 30 years	47	120	3.13(0.69 – 14.16)	
	Gov't employee	27	47	1.92(0.69 – 5.35)	
	Merchant	20	60	1.11(0.39 – 3.15)	
Occupation	Housewife	47	191	0.82(0.31 – 2.16)	***
	Other****	6	20	1	
Income	< 500 ETB	56	222	1	
	500 – 1000 ETB	10	33	1.20(0.56 – 2.58)	
	1001 – 1500 ETB	5	13	1.53(0.52 – 4.46)	***
	≥ 1501 ETB	29	50	2.30(1.34 – 4.00)	
	Not attend formal education	9	87	0.20(0.10 – 0.43)	**3.82(1.70 – 8.65)***
Educational status	Attend primary school	16	84	0.37(0.20 – 0.68)	2.12(1.10 – 4.01)
	Secondary school and above	75	147	1	1
Access for media	Yes	95	272	3.21(1.24 – 8.33)	
	No	5	46	1	***
	Primpara	25	105	0.64(0.35 – 1.20)	**2.31(1.13 – 4.71)**
Parity	Multipara	48	140	0.93(0.54 – 1.61)	***
	Grand multipara	27	73	1	**1**
	Yes	99	312	1.69(0.23 – 10.43)	***
ANC visit	No	1	6	1	
Frequency of ANC	≥4	82	201	1	**1**
	1 – 3	17	111	0.38(0.21 – 0.67)	**2.04(1.10 – 3.81)****
	Health institution	98	294	4.00(0.93 – 17.23)	
Place of birth	Home	2	24	1	***
Decision making power	1= self	54	73	1	**1**
	2= family and husband	46	245	0.25(0.16 – 0.41)	**3.68(2.21 - 6.11)***

~~*Statistically significant variable at p-value < 0.01; ** significant variable at p-value <0.05; ***Variables which are not statistically significant in multivariable logistic regression ****Students~~

~~-~~DISCUSSION

This study reported that 23.9% of the study participants were knowledgeable on postpartum complications.

This study finding is much higher than a similar study conducted in Western Bengal of the Indian state, in 2011, in which the percentage of mothers who were knowledgeable on postpartum complications ~~were~~ was 1.1% [15]. This difference could be due to variation in socio-demographical and time duration of the study.

The finding of a ~~cross~~ cross-sectional study carried out in rural area Gurage Zone, Ethiopia that reported 20.3% of mothers had mentioned two and above complications after ~~child birth~~ childbirth, but in this study, 23.9% of mothers had mentioned three and above postpartum complications [16]. This study discrepancy might be due to the difference of study areas.

This study finding is slightly similar ~~with~~ to ~~cross~~ cross-sectional studies conducted in two districts of the country; the Goba and Raya Kobo districts of Ethiopia that showed 22.1% & 26.4% of the mothers were knowledgeable on postpartum complications respectively [13, 14]. This consistence might be due to the similarity of the studies; in declaring mothers who had mentioned three and above complications after ~~child birth~~ childbirth as knowledgeable.

The study finding is also less as compared with studies conducted in different parts of Ethiopia, such as Aleta Wondo Sidama (37.7%), Fango district Wolita (55.8%), & Debre birhan Amhara (77.2%) [17-19]. This difference might be mothers who could mention two and above complications were considered as knowledgeable. But in the current study mothers were declared as knowledgeable if they mentioned three and above complications after ~~child birth~~ childbirth.

This study revealed that severe vaginal bleeding was the most commonly known postpartum complication mentioned by study participants followed by hypertension, breast complication & ~~foul~~ foul-smelling discharge. This result is slightly in line with different research findings conducted in other countries as well as in Ethiopia [20-22]. The least mentioned complication

was postpartum psychoses and blues which were never mentioned in other research findings. This might be due to maternal history of previous experience of the complication.

In this study; educational status, being grand multiparity, the number of ANC visits, ~~&~~ and decision-~~decision~~ making power of mothers to seek health care were predictors of maternal knowledge of postpartum complications.

~~Educational~~ The educational status of the mothers showed a statistical association with knowledge of postpartum complications. Women who attended secondary and above educational level were about 4 times (AOR = 3.82, 95% CI: [1.70, 8.65] more likely to be knowledgeable than mothers who didn't attend formal education. This was consistent with a research finding of Raya Kobo, Ethiopia [14].

Similarly, the frequency of antenatal care was another predictor of women's knowledge of postpartum complications. And women who had four and above ANC follow up were 2 times **(**AOR **=** 2.04, 95% CI: [1.10, 3.81]) more likely to be knowledgeable on postpartum complications than women who had three and ~~less~~ fewer ANC visits. This study finding is similar ~~with~~ to different studies conducted in Ethiopia [23, 24].

Being grand multiparity also has a relationship with maternal knowledge of postpartum complications. Grand multipara mothers were 2 times (AOR= 2.31, 95% CI: [1.13, 4.71]) more likely to have knowledge of complications of ~~child birth~~ cildbirth than primipara mothers. This is similar ~~with~~ to the ~~institutional~~ institutional-based ~~cross~~ cross-sectional (2014) study finding of Mechekel East Gojjam Zone, Ethiopia [25].

Another very important variable that was associated with maternal knowledge of postpartum complications, with a p-value of < 0.001 was the ~~decision~~ decision-making power of mothers to seek health service utilization. Mothers who decide health service utilization in their household had 4 times (AOR = 3.68, 95% CI: [2.21, 6.11]) a likelihood to be knowledgeable than mothers who depend on the decision of other family members.

This is in line with study findings conducted in Arba Minch town, Ethiopia; that test association for general knowledge of obstetrical complications of the three periods [24].

LIMITATION & STRENGTH OF THE STUDY

Due to the nature of ~~cross~~ the cross-sectional study design, this study couldn't establish cause and effect relationships this is the main limitation of the study.

As a strength, the study was conducted at the community level ~~that~~, which increases the generalizability to the entire urban area.

CONCLUSION AND RECOMMENDATIONS

Conclusion

According to this result mother's knowledge of postpartum complications is low. The most known complication after ~~child birth~~ childbirth ~~were~~ was severe vaginal bleeding followed by hypertension. Educational status, being grand multipara, the number of ANC visits, ~~&~~ and the ~~decision~~ decision-making power of mothers to seek health care were statistically associated variables that will predict maternal knowledge of postpartum complications.

Recommendations

Improving women's educational status and decision-making power to seek health care before complications happened is needed. Increasing information delivery system about specific postpartum complications during ANC follow-up are the recommended interventions.~~Encouraging women education and decision making power to seek health care before complications happened, and increasing information delivery system about specific postpartum complication during ANC follow up are the recommended interventions.~~ Based on the study findings the following concerned bodies should be recommended to improve and strengthen women's knowledge of postpartum complications.

- Health personnel and health extension workers should strengthen awareness creation activities by conducting maternal discussion forums at the community level.
- The Ministry of health should continue to encourage pregnant mothers to attend four and above antenatal visits.
- Health extension workers and community leaders should mobilize the society about the advantage of women's self decision-making power to seek health care at the household level.
- The Ministery of education Should continue to encourage women's education.

- Further research supported with qualitative data will be needed.
- ~~Some number of mothers had no information on postpartum complications, so health personals and health extension workers should strengthen their awareness creation activities.~~
- ~~The government should continue to strengthen and encourage mothers to attend ANC visit, decision making power to seek care and increasing their educational levels.~~
- ~~Further research using cohort methods will be needed that focused on knowledge of postpartum complications.~~

ACKNOWLEDGMENTS

Acknowledgment

We would like to acknowledge Arba Minch University College of Medicine and Health Sciences ~~in~~ for providing with necessary financial & material and making the work successful. We are thankful to all the participants who invested their time and perspectives with us.

REFERENCES

1. Health, F.M.o., *Management protocol on selected obstetrics topics*. 2010, Federal Democratic Republic of Ethiopia.
2. Huang, K., et al., *Does delivery mode affect women's postpartum quality of life in rural China?* Journal of clinical nursing, 2012. **21**(11- 12): p. 1534-1543.
3. Organization, W.H., *WHO recommendations on postnatal care of the mother and newborn*. 2014: World Health Organization.
4. Alkema, L., et al., *Global, Regional, and National Levels and Trends in Maternal Mortality Between 1990 and 2015, With Scenario-based Projections to 2030.* Obstetric Anesthesia Digest, 2016. **36**(4).
5. Nations, U., *The Sustainable Development Goals Report 2016.* United Nations: New York, NY, USA, 2016.
6. Organization, W.H., *Trends in maternal mortality: 1990-2015: estimates from WHO, UNICEF, UNFPA, World Bank Group, and the United Nations Population Division: executive summary*. 2015, World Health Organization.
7. Say, L., et al., *Global causes of maternal death: a WHO systematic analysis.* The Lancet Global Health, 2014. **2**(6): p. e323-e333.
8. Demographic, Z.Z., *Health Survey: Key Indicators.* Rockville, Maryland, 2016.
9. Nazik, E. and G. Eryilmaz, *The prevention and reduction of postpartum complications: Orem's Model.* Nursing science quarterly, 2013. **26**(4): p. 360-364.

10. Abdella, A., *Maternal mortality trend in Ethiopia.* Ethiopian Journal of Health Development, 2010. **24**(1).
11. Berhan, Y. and A. Berhan, *Commentary: reasons for persistently high maternal and perinatal mortalities in Ethiopia: part III–perspective of the "three delays" model.* Ethiopian ~~journal~~ Journal of health sciences, 2014. **24**: p. 137-148.
12. Office:, A.M.t.H., *Report of district health department;*. 2019.
13. Bogale, D. and D. Markos, *Knowledge of obstetric danger signs among child bearing age women in Goba district, Ethiopia: a cross-sectional study.* BMC pregnancy and childbirth, 2015. **15**(1): p. 77.
14. Bililign, N. and T. Mulatu, *Knowledge of obstetric danger signs and associated factors among ~~reproductive~~ reproductive-age women in Raya Kobo district of Ethiopia: A community based cross-sectional study.* BMC pregnancy and childbirth, 2017. **17**(1): p. 70.
15. Mukhopadhyay, D.K., et al., *Status of birth preparedness and complication readiness in Uttar Dinajpur District, West Bengal.* Indian Journal of public health, 2013. **57**(3): p. 147.
16. Zepre, K. and M. Kaba, *Birth preparedness and complication readiness among rural women of reproductive age in Abeshige district, Guraghe zone, SNNPR, Ethiopia.* International journal of women's health, 2017. **9**: p. 11.
17. Hailu, M., A. Gebremariam, and F. Alemseged, *Knowledge about obstetric danger signs among pregnant women in Aleta Wondo District, Sidama Zone, Southern Ethiopia.* Ethiopian ~~journal~~ Journal of health sciences, 2010. **20**(1).
18. Gebre, M., A. Gebremariam, and T.A. Abebe, *Birth preparedness and complication readiness among pregnant women in Duguna Fango District, Wolayta Zone, Ethiopia.* PloS one, 2015. **10**(9).
19. Akibu, M., *Prevalence and Associated Factors of Postnatal Care Utilization.* 2016.
20. Echoka, E., et al., *Barriers to emergency obstetric care services: accounts of survivors of ~~life~~ life-threatening obstetric complications in Malindi District, Kenya.* The Pan African medical journal, 2014. **17**(Suppl 1).
21. Ekabua, J.E., et al., *Awareness of birth preparedness and complication readiness in southeastern Nigeria.* ISRN obstetrics and gynecology, 2011. **2011**.
22. Karkee, R., et al., *The role of obstetric knowledge in utilization of delivery service in Nepal.* Health education research, 2014. **29**(6): p. 1041-1048.
23. Maseresha, N., K. Woldemichael, and L. Dube, *Knowledge of obstetric danger signs and associated factors among pregnant women in Erer district, Somali region, Ethiopia.* BMC women's health, 2016. **16**(1): p. 30.
24. Workineh, Y., et al., *Knowledge of obstetric danger signs and its associated factors in Arba Minch town, Ethiopia.* Am J Health Res, 2014. **2**(5): p. 255-9.
25. Amenu, G., et al., *Knowledge about danger signs of obstetric complications and associated factors among postnatal mothers of Mechekel District Health Centers, East Gojjam Zone, Northwest Ethiopia, 2014.* Scientifica, 2016. **2016**.

Response to reviewers

Dear academic editor and reviewers,

Thanks for the important ideas raised, which laid down a base for me to be a family of PLoS One in the future. And I am, so surprised and interested with your raised points. During revision, I have read PLOS ONE'S publication criteria, as well as the reviewed comments concerning the manuscript. In addition to my appreciation of PLOS ONE guidelines and your constructive review comments, I have explained some of my responses as follows.

A revised manuscript is prepared in two versions; **'Review Manuscript With Track changes'** and without tracked change which is labeled as **'Manuscript'**.

The funding statement and Data availability revisions are written on the cover letter to be approved.

Figures and tables are revised, based on the guideline of the journal.

Comments on grammatical and punctuation mistakes raised by academic editors and reviewers are more considered. Then, I have tried my best on comments throughout the documents during the revision process. Further editions are written and explained in detail within the revised document.

Additional Response To Comments

Introduction

'Delay I' is a contributing factor to maternal death in Ethiopia. This delay is due to a lack of mothers' knowledge of complications resulting in death. As explained, most maternal deaths have occurred after childbirth, which means in the postpartum period than antenatal and intra-natal periods. However, little has been done about maternal knowledge of postpartum complications. Therefore, this study tried to identify factors that influence maternal knowledge. The finding of the study improves maternal knowledge and increases early seeking of health care before life-threatening complications occurred.

Methods and Materials

Study Area and Period

The manuscript is revised according to the comment raised and other needed issues.

Population

Necessary revisions to the manuscript were done. Regarding the exclusion criteria which stated that mothers who were severely ill were excluded, does not mean they have poor knowledge. But due to the discomfort of the illness, they might not complete the interview. This might affect the quality of the data.

Sample size determination

The sampling formula was included in the revised manuscript. The expected percentage of mothers' knowledge of postpartum complications was properly cited.

A design effect of 1.5 was used to calculate the sample size. This is basically, quantifies the extent to which the expected sampling error in a survey departs from the sampling error that can be expected if simple random sampling was applied. For the question why not 2 or 2.5? design effect was not used in this specific study was economical feasibility and cost incurred to the study

Sampling Procedure

Revisions were done to the manuscript depending on the comments related to sampling procedures. As explained in the document the number of mothers who gave birth in each of Four (4) Kebeles were exactly different. But by chance, the rounded 'K-value' for all Kebeles was 3.

Operational definitions

A free chance to list the complications was given to the mothers and this increased the level of measurement. A single complication may result in the death of a mother. For example severe vaginal bleeding after childbirth. Therefore knowing at least three complications may alarm mothers to seek health care early and those mothers qualified to have adequate knowledge. The other thing was the purpose of comparison with related literature.

Data quality management

Comment: Please clarify on the (21)

Response: Pretesting of the questionnaire was carried out on 5% of the sample that was, on 21 mothers in Mirab Abaya town, Gamo Zone, Ethiopia.

Data processing and analysis

Comment: Please explain the rationale for the inclusion of variables with a P-value ≤ 0.25 instead of P-value ≤ 0.05 with relevant references. Also, clarify regarding the quality of the

previous studies that had variables with associations that were included in the multiple regression analysis.

Response: As per the scholarly recommendations, when a given variable is already proven to be related to the outcome of interest by reviewing previous literature on the subject and consulting experts in the field the purposeful selection is applied. According to Bendel RB, Afifi AA. (1977), and Mickey J, Greenland S(1989) any variable having a significant univariable test at some arbitrary level is selected as a candidate for the multivariable analysis. We base this on the Wald test from logistic regression and a p-value cut-off point of 0.25. More traditional levels such as 0.05 can fail in identifying variables known to be important (Bendel RB, Afifi AA. (1977) and Mickey J, Greenland S(1989)).

To this specific study, Model fitness was checked by Hosmer and Lemeshow's goodness of model fit test (P=0.434).

Ethics consideration

Comment to the ethics part was considered and revised accordingly. The written ethical letter was also uploaded as a supporting file with the manuscript.

Comments regarding, Results, Discussions, conclusions, and recommendations.

Comments raised by reviewers and editors under the result, discussions, conclusions, and recommendations were considered, and necessary revisions were done.

I look forward to receiving your acceptance response

Sincerely,

Godana, Principal author on behalf of other Co-authors
