# Supplementary material for: Knowledge of postpartum complications and associated factors among women who gave birth in the last 12 months in Arba Minch Town, Southern Ethiopia, 2019: A community-based cross-sectional study
Source: PLoS One. 2023 Feb 6;18(2):e0281242. doi: 10.1371/journal.pone.0281242 (PMC9901784; doi:10.1371/journal.pone.0281242)
Supplement: Attachment — Submitted filename: Review comments.docx [file pone.0281242.s002.docx]

Dear Editor,

Thank you for the opportunity to review this paper. I wish to congratulate the authors for this important study. However, I have some recommendations for the authors that will help improve the quality of the manuscript.

These comments are below;

I recommend the review of this manuscript by a proofreading service or by a fluent English researcher. It requires and extensive proofreading.

a. Copy editing needed to correct errors of spelling inconsistencies (e.g., household vs. house hold, P-value vs. p-value in sentences, the use of ‘&’ and ‘and’ interchangeably),

b. grammar e.g. Despite of several researches were conducted on maternal knowledge of obstetrics complications and associated factors, little is done on three separated phases of obstetrics i.e., pregnancy, child birth and postpartum periods.,

c. incomplete sentences e.g., Empowering women educational level, decision making power to seek health care and counseling during ANC follow up will needed. Empowering women educational level, decision making power to seek health care and counseling during ANC follow up will needed.

Introduction

1. ‘Delay I’ is the highest contributing factor of maternal mortality in Ethiopia and lack of knowledge on pregnancy related problem is the main issue result in ‘delay I…… Authors made a definite statement without backing it with literature.
2. Full of grammatical errors. Authors should correct all of them and punctuation mistakes throughout the work. E.g…. factors among women gave birth in the last 1year in Arba Minch Town, Ethiopia.

Materials and Methods

Sample size determination

Not clear whether the 22.1% knowledge about postpartum is an assumed value. Is the estimated 418 sample size rather making reference to another study, as indicated as “…. The required sample size was 418 [13].”

The 1.5 design effect is not also justified also a multi-stage strategy was employed. Why not 2 or 2.5?

Sampling procedure

If proportion to size allocation was employed, that means the number of the mothers in each Kebele should be different (if not significantly different). If that’s the case then the ‘K’-value (not defined) of 3, should not be constant for the four communities.

Ethical consideration

1. Authors should provide the reference for the ethical approval

Results

1. A total of 418 women who gave birth in the last twelve months preceding the study period were designed to participate in the study, this gives 100% response rate: I suggest they replace the ‘designed to participate’ with ‘recruited’ into the study.
2. The authors should provide the minimum and maximum age i.e age range.
3. I suggest the results should be re-written to make it clearer. The language employed does not communicate clearly
4. The other mentioned complications were breast complication 75(17.9%), blurred vision and weakness 73(17.5%), foul smelling vaginal discharge 71(17%), convulsions 14(3.3%) and the least mentioned was **postpartum psychoses and blues (please provide the frequency and percentages for postpartum psychoses and blues for consistency).**
5. Most of the respondents that had information on postpartum complications have interest to visit health institution if thus complications occurred. The main reason mentioned by mothers who had no any information about complications that could happen after child birth was never heard about any complications. (Please provide the figures for these as well)
6. Authors should interpret the results on the adjusted odd ratios. This will give more information to a person who does not understand numbers
7. Although stated in the methods the estimate from the Model fitness method, Hosmer and Lemeshow goodness of model fit test was not provided and explained.

Discussion

The Authors did not clearly discuss the study findings and its implications. For instance the findings of the factors which is the main objective was not clearly discussed. I suggest the Authors take their time to discuss the study findings well and extensively. They should clearly discuss the public health implications of the findings.

They should avoid repeating the results in the discussion section and rather focus on the implications on policy action and how the results can be used to address the gaps identified.

Limitation and strength

The limitation and strength should be discussed extensively.

Conclusion

The conclusion is loosely written

Recommendation

Authors should re-write the recommendation section. It should be based on the key findings.
