# Supplementary material for: Knowledge of postpartum complications and associated factors among women who gave birth in the last 12 months in Arba Minch Town, Southern Ethiopia, 2019: A community-based cross-sectional study
Source: PLoS One. 2023 Feb 6;18(2):e0281242. doi: 10.1371/journal.pone.0281242 (PMC9901784; doi:10.1371/journal.pone.0281242)
Supplement: Attachment — Submitted filename: Response to reviewers.docx [file pone.0281242.s003.docx]

**Response to reviewers**

Dear academic editor and reviewers,

Thanks for the important ideas raised, which laid down a base for me to be a family of PLoS One in the future. And I am, so surprised and interested with your raised points. During revision, I have read PLOS ONE’S publication criteria, as well as the reviewed comments concerning the manuscript. In addition to my appreciation of PLOS ONE guidelines and your constructive review comments, I have explained some of my responses as follows.

A revised manuscript is prepared in two versions; **‘Review Manuscript With Track changes’** and without tracked change which is labeled as **‘Manuscript’**.

The funding statement and Data availability revisions are written on the cover letter to be approved.

Figures and tables are revised, based on the guideline of the journal.

Comments on grammatical and punctuation mistakes raised by academic editors and reviewers are more considered. Then, I have tried my best on comments throughout the documents during the revision process. Further editions are written and explained in detail within the revised document.

**Additional Response To Comments**

**Introduction**

‘Delay I’ is a contributing factor to maternal death in Ethiopia. This delay is due to a lack of mothers’ knowledge of complications resulting in death. As explained, most maternal deaths have occurred after childbirth, which means in the postpartum period than antenatal and intra-natal periods. However, little has been done about maternal knowledge of postpartum complications. Therefore, this study tried to identify factors that influence maternal knowledge. The finding of the study improves maternal knowledge and increases early seeking of health care before life-threatening complications occurred.

**Methods and Materials**

**Study Area and Period**

The manuscript is revised according to the comment raised and other needed issues.

**Population**

Necessary revisions to the manuscript were done. Regarding the exclusion criteria which stated that mothers who were severely ill were excluded, does not mean they have poor knowledge. But due to the discomfort of the illness, they might not complete the interview. This might affect the quality of the data.

**Sample size determination**

The sampling formula was included in the revised manuscript. The expected percentage of mothers' knowledge of postpartum complications was properly cited.

A design effect of 1.5 was used to calculate the sample size. This is basically, quantifies the extent to which the expected sampling error in a survey departs from the sampling error that can be expected if simple random sampling was applied. For the question why not 2 or 2.5? design effect was not used in this specific study was economical feasibility and cost incurred to the study

**Sampling Procedure**

Revisions were done to the manuscript depending on the comments related to sampling procedures. As explained in the document the number of mothers who gave birth in each of Four (4 ) Kebeles were exactly different. But by chance, the rounded ‘K-value’ for all Kebeles was 3.

**Operational definitions**

A free chance to list the complications was given to the mothers and this increased the level of measurement. A single complication may result in the death of a mother. For example severe vaginal bleeding after childbirth. Therefore knowing at least three complications may alarm mothers to seek health care early and those mothers qualified to have adequate knowledge. The other thing was the purpose of comparison with related literature.

**Data quality management**

Comment: Please clarify on the (21)

Response: Pretesting of the questionnaire was carried out on 5% of the sample that was, on 21 mothers in Mirab Abaya town, Gamo Zone, Ethiopia.

**Data processing and analysis**

**Comment**: Please explain the rationale for the inclusion of variables with a P-value ≤ 0.25 instead of P-value ≤ 0.05 with relevant references. Also, clarify regarding the quality of the previous studies that had variables with associations that were included in the multiple regression analysis.

**Response:** As per the scholarly recommendations, when a given variable is already proven to be related to the outcome of interest by reviewing previous literature on the subject and consulting experts in the field the purposeful selection is applied. According to Bendel RB, Afifi AA. (1977), and Mickey J, Greenland S(1989) any variable having a significant univariable test at some arbitrary level is selected as a candidate for the multivariable analysis. We base this on the Wald test from logistic regression and a p-value cut-off point of 0.25. More traditional levels such as 0.05 can fail in identifying variables known to be important (Bendel RB, Afifi AA. (1977) and Mickey J, Greenland S(1989)).

To this specific study, Model fitness was checked by Hosmer and Lemeshow's goodness of model fit test (P=0.434).

**Ethics consideration**

Comment to the ethics part was considered and revised accordingly. The written ethical letter was also uploaded as a supporting file with the manuscript.

**Comments regarding, Results, Discussions, conclusions, and recommendations.**

Comments raised by reviewers and editors under the result, discussions, conclusions, and recommendations were considered, and necessary revisions were done.

**I look forward to receiving your acceptance response**

**Sincerely,**

**Godana, Principal author on behalf of other Co-authors**
