# Supplementary material for: Knowledge of postpartum complications and associated factors among women who gave birth in the last 12 months in Arba Minch Town, Southern Ethiopia, 2019: A community-based cross-sectional study
Source: PLoS One. 2023 Feb 6;18(2):e0281242. doi: 10.1371/journal.pone.0281242 (PMC9901784; doi:10.1371/journal.pone.0281242)
Supplement: Attachment — Submitted filename: Response to the reviewers.docx [file pone.0281242.s007.docx]

**Response to reviewers**

Dear academic editor and reviewers,

Thanks for your extensive and constructive comments. I am so happy and interested in all your review prosses. In addition to my appreciation of PLOS ONE guidelines and your constructive review comments, I have explained some of my responses as follows.

A revised manuscript is prepared in two versions; **‘Review Manuscript With Track changes’** and without tracked change which is labeled as **‘Manuscript’**.

Comments regarding the English language part were raised during the review prosses and, I have done it accordingly throughout the whole document.

Additionally, the reference section was revised and through the online reference importing system, all references are written according to the Vancouver referencing style. I have seen the reference written to the ethical approval letter which is not nessessory and already removed from the manuscript. Then, I tried my best on comments throughout the documents during the revision process. Further editions are written and explained in detail within the revised document.

**I look forward to receiving your acceptance response**

**Sincerely,**

**Godana, Principal author on behalf of other Co-authors**
